# Symmetry‐Imposed Selection Rules for Excitations of Nontrivial Plasmonic Topologies

**DOI:** 10.1002/advs.77043

**Published:** 2026-08-10

**Authors:** Jie Yang, Pengyi Feng, Fei Han, Weijin Chen, Zhongwei Jin, Jincheng Ni, Yuanjie Yang, Anxue Zhang, Guy A. E. Vandenbosch, Niels Verellen, Ewald Janssens, Jiafu Wang, Benfeng Bai, Cheng‐Wei Qiu, Xuezhi Zheng

**Affiliations:** ^1^ Shaanxi Key Laboratory of Artificially‐Structured Functional Materials and Devices Xi'an China; ^2^ Changchun Institute of Optics Fine Mechanics and Physics Chinese Academy of Sciences Changchun China; ^3^ WaveCoRE research group KU Leuven Leuven Belgium; ^4^ State Key Laboratory of Precision Measurement Technology and Instruments Department of Precision Instrument Tsinghua University Beijing China; ^5^ Quantum Solid‐State Physics Department of Physics and Astronomy KU Leuven Leuven Belgium; ^6^ IMEC Leuven Belgium; ^7^ Department of Electrical and Computer Engineering National University of Singapore Singapore Singapore; ^8^ College of Optical and Electronic Technology China Jiliang University Hangzhou China; ^9^ School of Physics University of Electronic Science and Technology of China Chengdu China; ^10^ Xi'an Jiaotong University Xi'an Shaanxi China; ^11^ Suzhou Laboratory Suzhou China; ^12^ College of Electronic and Information Engineering Nanjing University of Aeronautics and Astronautics Nanjing Jiangsu China

**Keywords:** nearfield topology, selection rule, singularity, symmetry, topological quasiparticle

## Abstract

Nontrivial nearfield topologies in nano‐optics refer to nearfield configurations embedded within singularities or topological defects, providing an ideal platform to explore integrated optoelectronics and higher‐dimensional topological physics. Exciting such field topologies relies on selection rules related to various conserved quantities. Unfortunately, existing algebraic rules focus primarily on scalar singularities in nano‐optical (e.g., plasmonic) systems and largely neglect the vectorial nature of the fields. More critically, these rules remain phenomenological. Given the intrinsic link between conserved quantities and symmetries, here we establish a unified selection rule using group theory that govern the excitations of nontrivial field topologies across three photonic spin states in generic nanophotonic systems. This rule can act as building blocks for constructing selection rules for exciting and engineering higher‐dimensional field topologies (embedded within vectorial singularities and quasiparticles). These rules are derived purely from symmetry arguments and are therefore rooted in first principles. The proposed rules further predict two novel physical effects in plasmonic systems: spin‐orbit splitting of vortices and multidimensional nested vortices. Phase‐resolved in‐situ measurements of nested multidimensional plasmonic topologies well demonstrate our findings. Our group‐theory‐based approach can serve as a versatile framework for engineering symmetry‐ and singularity‐related phenomena—like circular meron lattices and plasmonic quasicrystals—in diverse wave systems.

## Graphical abstract

A unified group‐theory selection rule governs the excitation of vectorial nearfield topologies across three plasmonic spin states. Derived from first principles, it predicts spin–orbit vortex splitting and multidimensional nested vortices, confirmed by phase‐resolved in situ measurements. This symmetry framework enables the design of multidimensional nontrivial nearfield topologies in nano‐optics.

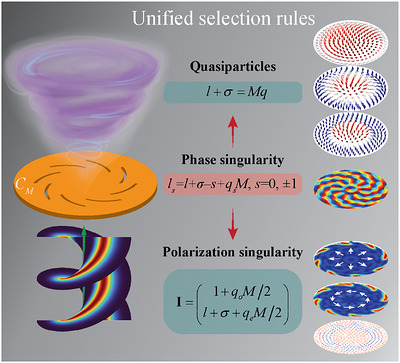

## 1 Introduction

Real‐space topologies of nearfields in nano‐optics are featured by the embedded singularities where physical quantities describing the fields are undetermined [1]. These singularities define the connectivity of field configurations and thus can be viewed as topological defects. As a representative nanophotonic system, plasmonic systems supporting surface plasmon polaritons (SPPs) have been explored to realize rich nontrivial nearfield topologies. Like singularities in generic wave fields [2], plasmonic singularities can be classified by dimension into two fundamental categories: scalar phase singularities and vectorial polarization singularities [3, 4]. Mixing the two types of singularities, higher‐dimensional singularities can be synthesized, e.g., 3D singularities like topological quasiparticles [1, 5, 6]. Multidimensional singularities give birth to a special set of SPP fields, plasmonic vortices across dimensions, exhibiting novel geometric and physical properties, such as highly localized and surface‐confined [7, 8], nontrivial of real‐space and spin‐texture topologies [5, 6, 9], and capable of carrying orbital angular momentum (OAM) on the nanometer and femtosecond scales [6, 7, 10, 11], and thus opening avenues to explore on‐chip optoelectronics [12, 13, 14, 15] and higher‐dimensional topological physics [16].

Excitation of nontrivial plasmonic topologies relies on plasmonic spin‐orbit interaction (SOI), which mixes spins and orbits of light and plasmons together [3, 7, 8]. Like physical processes in quantum and classical systems, plasmonic SOI is governed by selection rules that are strictly imposed by various symmetries and are strongly related to conserved quantities in plasmonic systems [17, 18, 19]. The first milestone of the rules emerged in 2008 with the discovery of a spin‐based plasmonic effect, demonstrating spin‐to‐orbital angular momentum transfer in plasmonic fields [8]. In 2010, rotational symmetry considerations introduced a so‐called geometric charge into the first rule, generalizing it to enable plasmonic vortices with arbitrary higher topological charges [3, 7, 10]. Later, advances in photonic OAM incorporate light's spin and orbital degrees of freedom into plasmonic SOI studies, broadening the rules to include light orbits [20, 21]. This leads to discoveries like plasmonic circular/helical dichroism [22, 23] and optical transitions beyond electric dipole interactions [21, 24]. In 2020, analytical SPP field studies further generalized these rules, predicting novel deuterogenic and nested plasmonic vortices and enabling subsequent kaleidoscopic on‐chip vortex beam generations [11, 25, 26]. However, these rules are only phenomenological and treat plasmonic fields as scalar fields, largely ignoring their vector nature and thus capturing only partial aspects of plasmonic SOI. Later work incorporating vector properties reveals algebraic rules for polarization singularities (V‐points, C‐points, L‐lines) in transverse SPP fields, uncovering effects like helicity locking and polarization vortices [3, 4]. Yet, these rules neglect light's orbital role and more critically, remain phenomenological.

Given the intrinsic link between selection rules and symmetries in various physical systems [17, 19], here, from a pure symmetry perspective we develop a generalized symmetry‐singularity interplay theory to formulate the excitations of nontrivial plasmonic nearfield topologies. By employing projection operator techniques in group theory, we first partition two Hilbert spaces spanned by vectorial eigenstates of plasmonic systems and incident light with spins and orbits, into two sets of complete and orthogonal subspaces, respectively, which are uniquely labeled by a set of irreducible representation (irrep) indices given by group theory. Then by matching the same irrep index, the spins and orbits of light and plasmons are linked together, thus giving birth to a fundamental selection rule which properly considers the vector nature and primarily governs the excitation of nontrivial plasmonic topologies across three plasmonic spin states. Building on the rule, we establish two selection rules for exciting higher‐dimensional plasmonic topologies (embedded within vectorial singularities and quasiparticles like skyrmions or merons). Rooted in first principles, these rules unify prior algebraic ones as special cases, and predict: 1) spin‐orbit splitting effect and 2) multidimensional nested plasmonic vortices, further revealing transformation among different configurations of quasiparticles and even circular meron lattices which are previously observed mostly in the form of periodic lattices or clusters [6, 27]. These rules are experimentally validated by using a homemade spin‐selective phase‐resolved SNOM (SSPR‐SNOM). Our findings also reflect peculiar spin and nonzero rest‐mass properties of SPPs as bosonic quasiparticles in the context of quantum electrodynamics [28]. These principles extend beyond plasmonics to other wave systems for studying multidimensional singularity phenomena, free‐space OAM spectrum engineering [29], moiré skyrmion lattices [30], plasmonic quasicrystals [16], and whispering gallery modes [31, 32], to name a few.

## 2 Symmetry‐Imposed Selection Rules

**FIGURE 1:**
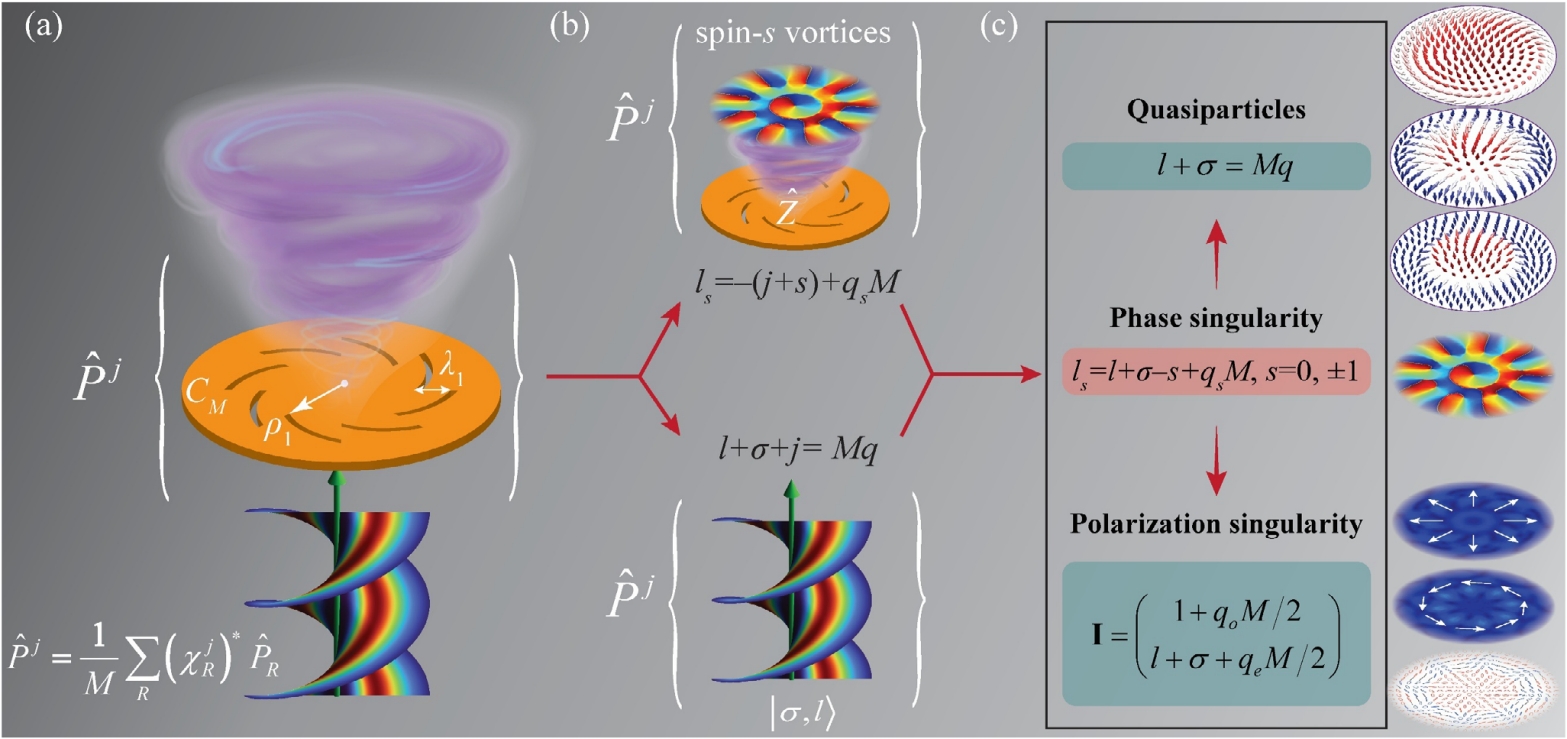
Symmetry‐imposed unified selection rules for exciting nontrivial plasmonic topologies: (a) Plasmonic SOI process, where ${\hat{P}}^{j}$ denotes the projection operator acting on the process; (b) Scheme of symmetry‐driven space partition approach via projection operator techniques, where ***j*** is the irrep index, as well as the orbital index of plasmonic vortices; (c) Obtained unified selection rules for exciting multidimensional plasmonic topologies, including scalar phase singularity, vectorial polarization singularities and three 3D singularities (including Bloch meron, Néel and bubble skyrmions).

Plasmonic SOI creating nontrivial nano‐nearfield topologies embedded within singularities (see Figure 1a) can be formulated into an operator equation within the framework of classic light‐matter interaction. The operator equation reads $\hat{Z}\left|\;J\rangle =|\sigma ,l\right\rangle$(see derivations in SI 1), where $\hat{Z}$ is the impedance operator that contains all eigen information of a plasmonic system; |σ, *l*〉 is the incident light that carries spin angular momentum of σℏ and OAM of *l*ℏ; and |*J*〉 denotes the currents excited by |σ, *l*〉. The plasmonic SOI can be investigated from three aspects: (1) the eigenstates of a plasmonic system; (2) the incident light; and (3) the excitation of eigenstates. For the first aspect, considering the eigenvalue problem of $\hat{Z}$ operator, we can obtain all eigenstates of the plasmonic system (i.e., eigen SPPs). These eigenstates span a Hilbert space under the definition of inner product. For the second aspect, considering the orthogonal relation $\left\langle \sigma_{1},l_{1}|\sigma_{2},l_{2}\right\rangle =\delta_{\sigma_{1},\sigma_{2}}\;\delta_{l_{1},l_{2}}$, the incident light |σ, *l*〉 also spans a Hilbert space after iterating over all possible combinations of σ and *l* (σ  =   ± 1 and l∈Z). For the third aspect, the SPPs excited by |σ, *l*〉 are weighted superposition of all eigenstates and conventionally need be preciously solved according to the geometric details of the plasmonic system. Typically, the plasmonic systems producing plasmonic singularities possess inherent symmetries, enabling to reformulate and simplify the plasmonic SOI with symmetry principles.

Figure 1a depicts a typical plasmonic system producing nontrivial plasmonic topologies, i.e., a plasmonic vortex lens (PVL). The PVL consists of eight spiral slits (the slit width is 200 nm) etched into a 105 nm thick gold film over a silica substrate. The inner radius of the eight spiral slits is described as ρ  = ρ_1_  + *M*φλ_1_/2π − (*a* − 1)λ_1_, where a=1,2,…,M, ρ_1_ =  3.1λ_1_ denotes the inner radius of the PVL, and λ_1_ denote the inner radius of the PVL and is set as the wavelength of SPPs. The PVL holds *M*‐fold rotational symmetries (*M*  =  8 Figure 1a) [7, 11]. These symmetries form a *C_M_* group. As an abelian group, the group defines *M* 1D irreducible representations (irreps which are indexed by an integer *j* ranging from 0 to *M* − 1, see Table S1 for all irreps of the *C_M_* group in SI 1) [17]. Owing to the commutative relations between the impedance operator $\hat{Z}$ and the symmetry operators of symmetry group *C_M_*, we can employ projection operator techniques offered by group theory to treat the three aspects of the plasmonic SOI in the PVL i.e., ${\hat{P}}^{j}\hat{Z}|\;J\rangle ={\hat{P}}^{j}\;\left|\sigma ,l\right\rangle$, where *j* denotes the irrep index and ${\hat{P}}^{j}$ denotes the projection operator of *j*‐th irrep (see Section S1).

Employing projection operator techniques to the first aspect, we can partition the Hilbert space spanned by the eigenstates of the PVL into a set of complete and orthogonal subspaces. Each subspace is uniquely labeled by an irrep index given by group theory and represents a category of eigen plasmonic vortices which can be expressed in terms of circular bases, corresponding to three polarization states, *E_z_* (*z* or out‐of‐plane plasmonic field), *E*_+_ and *E*_−_ (left‐ and right‐circular plasmonic field). Each polarization state exhibits a pattern of scalar vortices which hold topological charges (see Figure 1b) [33],

(1) $$l_{s}=-\left(j+s\right)+Mq_{s}$$

In the above, *j* is the irrep index; *s* takes 0, −1, and 1, representing three orthogonal polarization states of vectorial SPP fields (i.e., *z*, right‐ and left‐circular polarization states), respectively; *l_s_* denotes the topological charge of a scalar vortex in the polarization‐*s* state; *q_s_* denotes an arbitrary integer associated with the polarization‐*s* state.

(2) l+σ+j=Mq

where *q* is an arbitrary integer. Then, for the third aspect, we can apply the symmetry matching condition to discuss the excitation of eigen vortices, namely, the symmetries of the eigen vortices and the incident Laguerre–Gaussian (LG) modes should match with each other (see Section S1). In other words, in our case they should belong to the same irrep. Hence, cancelling the irrep index *j* in above two equations, we derive a fundamental symmetry‐imposed selection rule for exciting phase singularities in three polarization states of SPPs,

(3) $$l_{s}=l+\sigma -s+Mq_{s}$$

where *s* can be naturally identified as the spin quantum number of SPPs: 1) when *s*  =   ± 1, the plasmonic vortex is left/right‐circularly polarized and, accordingly, its spin quantum number is ± 1; 2) when *s*  =  0, the plasmonic vortex polarizes along *z* axis and, accordingly, its spin quantum number is 0. When *s*  =  0, our obtained selection rule degrades into the well‐known algebraic rules reported before [7, 10, 11, 21]. When *s*  =   ± 1, our obtained selection rule can be applied to explain the generation of cross‑polarized plasmonic scalar vortices under the analysis framework of geometric phase [26]. Note that the co/cross‑polarized SPP components in the previous works should be linked with ours by considering σ*s*  =   ± 1, respectively. The above symmetry‐imposed selection rule considers all spin states of SPPs and thus reflects the vector nature of SPPs. They further predict two things: (1) spin‐orbit splitting effect of plasmonic vortices and 2) multidimensional nested plasmonic vortices. For the first, from Equation (3) it can be observed that *l_s_* is spin‐dependent/locked (i.e., the spins of plasmonic vortices affects and controls their orbits) [34, 35]. For the second, it is known that the plasmonic scalar vortex in spin‐0 state can exhibit the deuterogenic plasmonic vortex at the central region, thus carrying multiplexed OAMs or multiple topological charges instead of single one [11, 25]. Equation (3) suggests that the nested plasmonic vortices exist not only in spin‐0 state, but also in spin‐±1 states and even in polarization vortices. The two predictions will be numerically and experimentally verified subsequently.

Finally, by extending the consideration from a discrete rotational group, *C_M_*, to a continuous U(1) Lie group, where the PVL holds continuous rotational symmetries [9, 26, 36], the selection rule in Equation (3) can be cast as, *l_s_* =  *l* + σ − *s* (see details in Section S3). Such the selection rule is nothing else than the well‐accepted conservation law of total angular momenta in continuously rotational physical systems given by Noether's theorem. In plasmonic systems, this rule indicates that for such PVL there also exists the spin‐orbit splitting effect, but no nested vortices in each spin state. The spin‐orbit splitting effect revealed here well explains the topological charges of spin‐selectively‐excited plasmonic singularities reported recently [36]. It is worth noting that our developed theoretical framework combines group‐theoretic symmetry analysis with a general electromagnetic interaction formalism, i.e., the electric field integral equation method, both of which are broadly general tools. As a result, the framework is applicable to essentially any classical electromagnetic or optical structure that possesses well‐defined geometrical symmetries, provided quantum effects are negligible (see some examples in Figure S1).

## 3 Nested Plasmonic Singularities and Vortices

### 3.1 Nested Phase Singularities and Scalar Vortices

**FIGURE 2:**
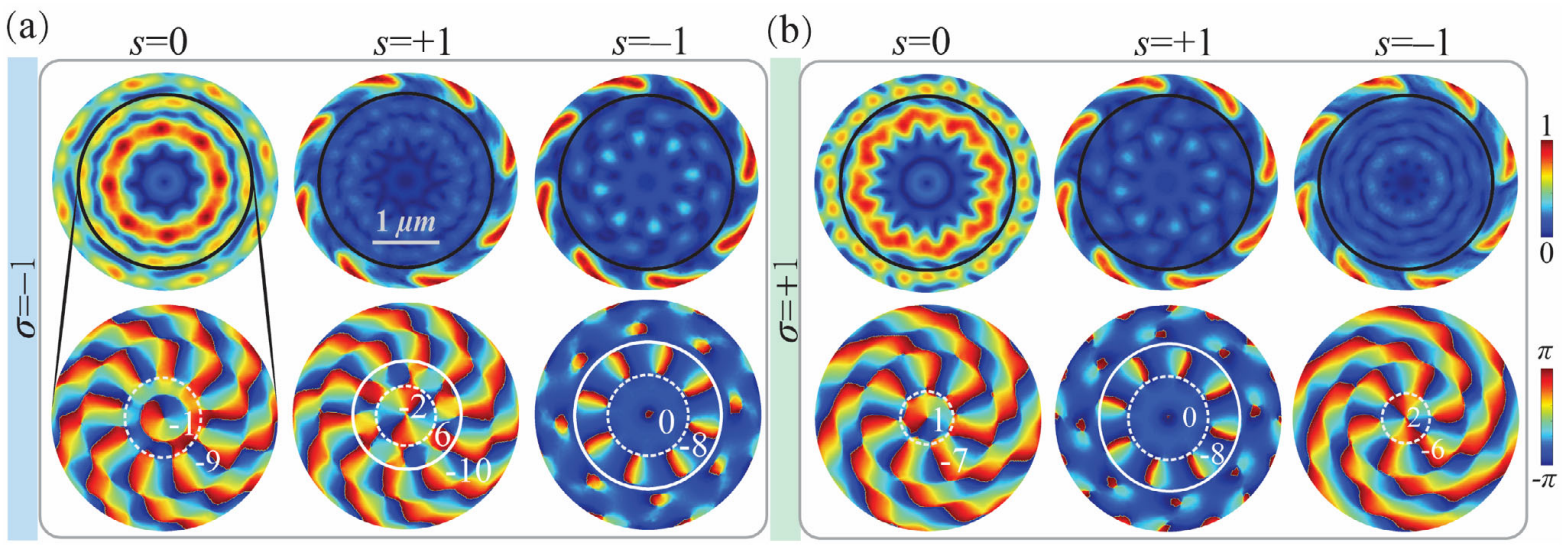
Nested plasmonic singularities and scalar vortices in three spin states: (a) and (b) depict the magnitude and phase patterns of plasmonic scalar vortices in three spin states excited by | −, 0〉 and | +, 0〉 (denoted by ***σ *** = * *∓1 in the plot, respectively). In the phase patterns, nested plasmonic vortices in three spin states are delineated by white lines and their topologies charges ***l***_***s***_ are marked by white numbers.

To verify our proposed selection rule, a PVL with eight‐fold rotational symmetries is designed (see Figure 1a). The incident field |σ, *l*〉 is shone from the back of the PVL, whose wavelength is chosen as 633 nm. Accordingly, the SPP wavelength is about 606nm [25]. The PVL is illuminated by the left‐ and right‐circularly polarized beams (i.e., | +, 0〉 and | −, 0〉), separately. The theoretical discussions suggest that the PVL generate plasmonic scalar vortices in three spin states and the vortices possibly carry topological charges determined by Equation (3), that is, for the case of | +, 0〉, *l_s_* =  1 − *s* + 8*q_s_*; and for the case of | −, 0〉, *l_s_* =   − 1 − *s* + 8*q_s_*. To verify, COMSOL Multiphysics is used to simulate the responses of the PVL under two illuminations. The simulated SPP fields in three spin states are illustrated in Figure 2, from which it can be observed that the SPP fields in different spin states indeed form different plasmonic scalar vortices, well demonstrating our previous two predictions. Take the case of the | −, 0〉 incidence as an example (see Figure 2a). For the first prediction, from Figure 2a it can be observed that the topological charges *l_s_* differ from the related spin state, i.e., spin dependence. For the second one, from Figure 2a it can be observed that for a certain spin state, the topological charges carried by the plasmonic scalar vortex are a nest of multiple scalar vortices with different topological charges and the deuterogenic scalar vortices always exist, i.e., the nested plasmonic singularities and scalar vortices are generated. The two predictions can also be demonstrated by the plasmonic scalar vortices belonging to the rest irreps of *C*_8_ group, which can be exci Figure S2 ed by different incident beams |σ, *l*〉 by properly choosing σ and *l* (see Figures S2 and S3 and S4 in Section S4).

### 3.2 Nested Polarization Singularities and Vector Vortices

Next, we take the above fundamental selection rule as a “building bock” to construct the selection rule for exciting vectorial polarization singularities of SPPs, such as V points, C points, and L lines, accompanying with plasmonic polarization vortices. A generic polarization vortex is an inhomogeneous polarization pattern with polarization singularities on a manifold, which is the vectorial field instead of the scalar and can be understood from the perspective of superposition of two scalar vortices on the circular basis, i.e., a polarization can be expressed as | +, *l*_+_〉 + | −, *l*_−_〉 (see SI 5) [2, 37]. The topological invariant of the polarization vortex can be characterized by a topological charge vector **I**  =  (*I_o_*,*I_e_*), where *I_o_* and *I_e_* represent the geometric and dynamic topological charges carried by the polarization vortex, respectively. The two topological charges can be evaluated as Io=12πIm∮⟨e|∇|e⟩·dr and Ie=14πIm∮⟨Φ|∇|Φ⟩·dr along the closed contours encircling the singularity, where **e** is the normalized Jones vector and Φ  =  2*E*_+_ *E*_−_ is a so‐called dynamic scalar field [37]. There are simple algebraic connections between (*I_o_*,*I_e_*) and (*l*_−_,*l*_+_), namely, *I_o_* = (*l*_−_ − *l*_+_)/2 and *I_e_* = (*l*_−_ + *l*_+_)/2  [2, 37]. Our obtained selection rule in Equation (3) has revealed the topological charges of scalar vortices with phase singularities in spin‐±1 states. This naturally leads to the formation of plasmonic polarization singularities and vortices in SPPs. With the fundamental selection rule in Equation (3), the topological charge vector of plasmonic polarization singularities can be evaluated as,

(4) $$I=\left(\begin{array}{c} 1+\frac{q_{o}M}{2}\\ l+\sigma +\frac{q_{e}M}{2} \end{array}\right)$$

In the above, *q_o_* = *q*_−_  − *q*_+_ and *q_e_* = *q*_−_  + *q*_+_, where *q*_−_ and *q*_+_ represent the integers in plasmonic scalar vortices in spin‐∓1 states. The above equation serves as a general vectorial selection rule for exciting plasmonic polarization singularities and vortices in SPPs, further indicating that the polarization vortices also include the deuterogenic polarization vortices at the central region and carry multiple topological charges since *q*_−_ and *q*_+_ taking arbitrary integers in principle. In Equation (4), different components of **I** leads to different types of polarization singularities. For example, when *I_o_* always equals 1 and *I_e_* =  *l* + σ + *q_e_M*/2, then the arising polarization singularities are a V point occurring in the purely linearly polarized fields, e.g., in the radially and azimuthally polarized fields *E*_ρ_ and *E*_φ_ (see details in Sections S5 and S6); and when *I_o_* takes multiple values, much more complex polarization singularities like the multivalued C points appear.

**FIGURE 3:**
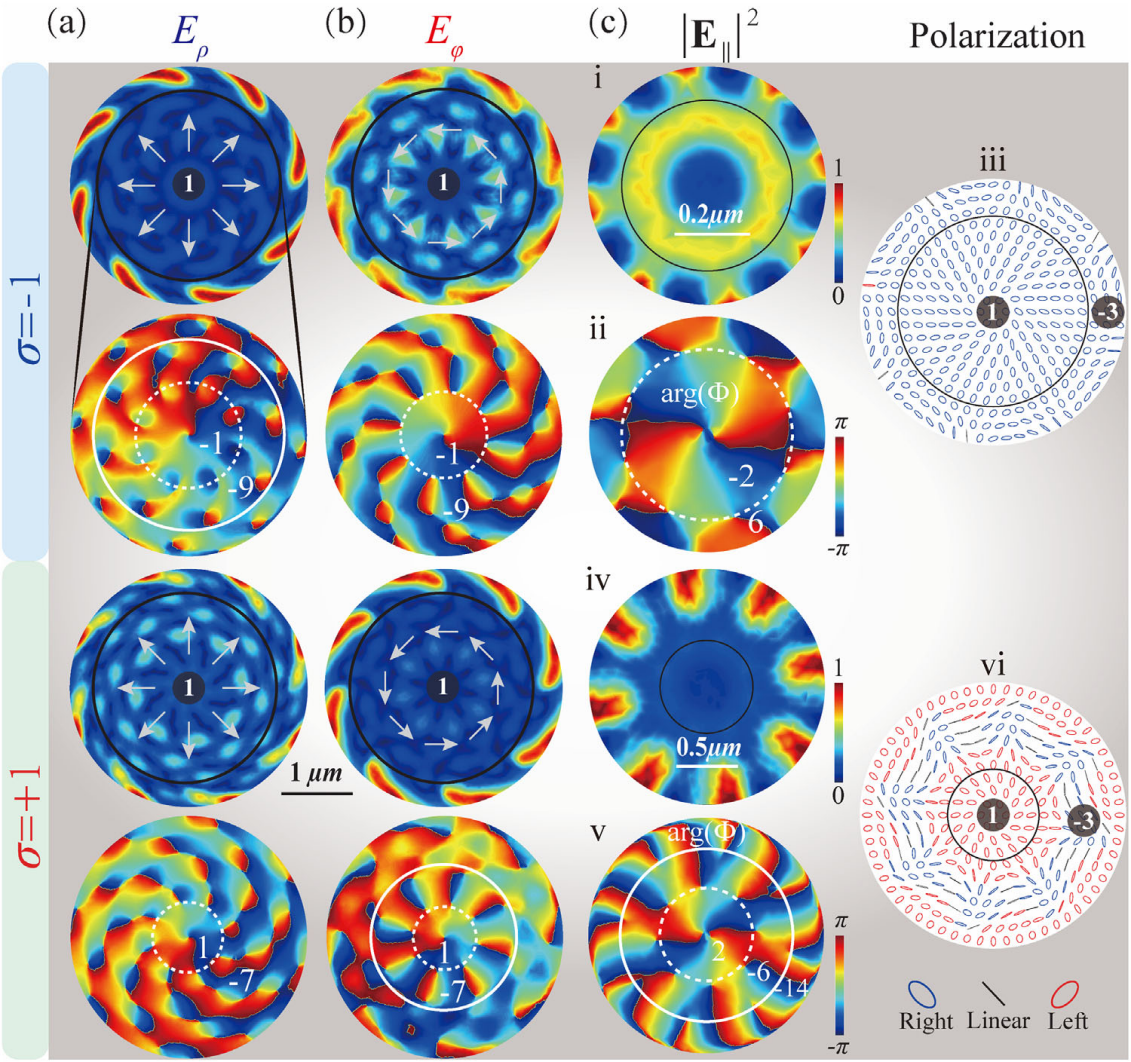
Nested plasmonic polarization singularities and vector vortices excited by | −, 0〉 and | +, 0〉, respectively: (a) V points in the radial polarized plasmonic field, (b) V points in the azimuthally polarized plasmonic field, (c) right‐ and left‐handed C points in the transvers SPP fields. In (a) and (b), the first and third rows correspond to the magnitude distributions of *E*_ρ_ and *E*_φ_, in which the grey vectors depict the local polarization states, and the geometric topological charges *I_o_* carried by V points in *E*_ρ_ and *E*_φ_ are marked with white numbers in black circles. The second and fourth rows correspond to the dynamic phase distributions of *E*_ρ_ and *E*_φ_, in which dynamic topological charges *I_e_* of singularities are marked by white numbers. In (c), i and iv illustrate the intensity distributions of transverse SPP fields (i.e., ${|E_{\left|\right|}|}^{2}=E_{x}^{2}+E_{y}^{2}$); ii and v illustrate the phase distributions of the auxiliary dynamic scalar field Φ; and iii and vi illustrate the polarization distributions of the in‐plane SPP fields, in which the geometric topological charges *I_o_* are marked by white numbers in black circles. In ii and v, white numbers are used to mark the multiple topological charges carried by the scalar vortices in Φ, which are evaluated by a line integral 12πIm∮⟨Φ|∇|Φ⟩·dr=2Ie.

Again, take the PVL in Figure 1a as an example to verify the vectorial selection rule. Figure 3 illustrates the plasmonic polarization vortices excited by | −, 0〉 and | +, 0〉. From Figure 3a,b, it can be observed that the geometric topological charges *I_o_* carried by the polarization vortices in *E*_ρ_ and *E*_φ_ are always one [38], and dynamic ones *I_e_* are multiple and agree well with our obtained selection rule in Equation (4). The polarization singularities in Figure 3a,b are V points. For the more complex polarization singularities in the transverse SPP fields, let's take the case of | −, 0〉 as an example. In Figure 3c(iii), a black circle divides the polarization distribution into two parts: an inner and an outer part. In the inner (outer) part, the polarization distribution exhibits the morphology of a polarization vortex with a C point at the center, whose geometric topological charge is 1 (−3). The geometric topological charges can be further confirmed by a so‐called Stokes field (see Figure S5 in Section S5). In Figure 3c(ii), a white dashed circle divides the phase distribution of the dynamic scalar field Φ into two parts: an inner and an outer part, carrying topological charges of −2 and 6, respectively. Since the dynamic topological charge *I_e_* carried by a polarization vortex is exactly equal to a half of the topological charge carried by the related scalar vortex in Φ (evaluated by 12πIm∮⟨Φ|∇|Φ⟩·dr), then *I_e_* corresponding to the polarization vortex in Figure 3c(iii) should be −1 and 3 in the inner and outer parts, respectively. Therefore, the polarization vortex in Figure 3c(iii) is a nest of two polarization vortices with different geometric and dynamic topological charges, i.e., a nested plasmonic polarization vortex, and the inner polarization vortex is a deuterogenic polarization vortex. The same story applies to the case of | +, 0〉 in Figure 3c(iv–vi). Similarly, the nested plasmonic polarization vortices can also be demonstrated existing in the radial, azimuthal, and in‐plane SPP fields belonging to the rest irreps of *C*_8_ group, which can be excited by different incident beams |σ, *l*〉 by properly tuning σ and *l* according to Equations (2) and (3) (see Figures S6 and S7 and S8 and S9 in Section S5). The above results well demonstrate the vectorial selection rule in Equation (4).

## 4 In Situ Observations of Nested Multidimensional Plasmonic Topologies

**FIGURE 4:**
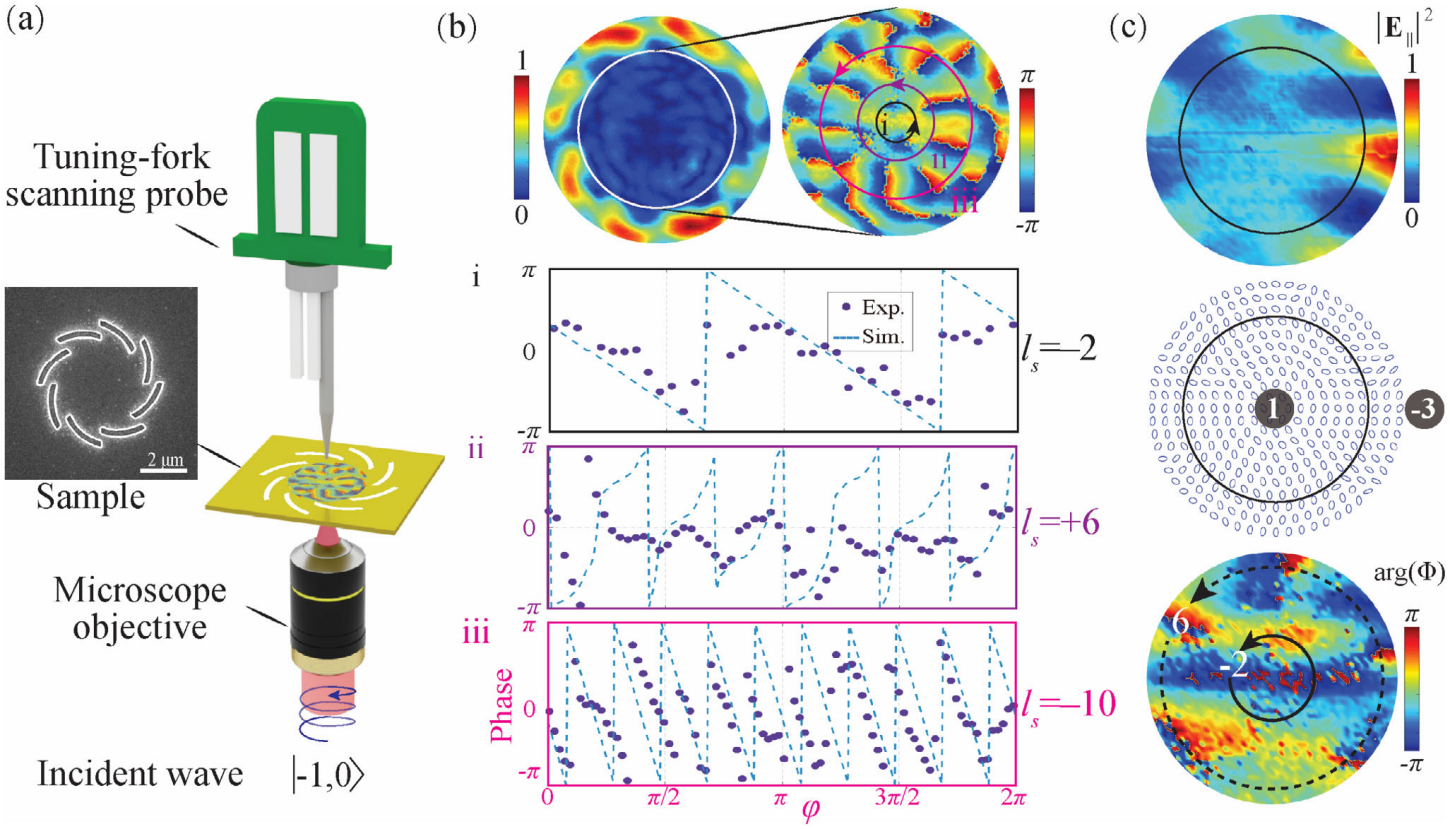
Observation of multidimensional plasmonic vortices excited by | −, 0〉: (a) Nearfield scanning setup with a aluminum‐coated aperture‐type tuning‐fork scanning probe, and scanning electron microscope image of the fabricated sample; (b) the measured magnitude and phase distributions of scalar vortex in spin‐+1 state (in comparison to the column denoted by ***s *** = * * + 1 in Figure 2a); (c) the measured polarization vortex (in comparison to Figure 3c(i–iii)). In (b), (i), (ii), and (iii) plot the simulated and experimental phase distributions of the vortex along three closed contours denoted by i, ii, and ii in the phase distribution of (b), respectively.

To observe the plasmonic singularities and vortices across dimensions and to experimentally verify our theory, a PVL sample is fabricated using a standard electron‐beam lithography (EBL) and reactive ion etching (RIE) process on a glass substrate (see Figure 4a below and Figure S10 in Section S7). The sample is illuminated by | −, 0〉 at the wavelength of 633 nm. A homemade in situ SSPR‐SNOM with subwavelength resolution in 3D space (see Figure S11 in Section S7) is used to characterize the excited SPP field in the near‐field region (see Figure 4a and methods). The near‐field amplitude and phase were directly measured through a heterodyne interferometric detection scheme. Specifically, the interference signal was demodulated by a lock‐in amplifier, from which the amplitude and phase were directly read out. Therefore, our experimental results are direct measurements, i.e., in situ observations, of the optical field, and the presented field images in Figure 4a were generated only by plotting the measured amplitude and phase without any Fourier‐domain filtering or reconstruction.

The measured scalar vortices in spin‐+ 1 state are visualized in Figure 4b, corresponding to the simulated results marked by *s*  =   + 1 in Figure 2a. It can be observed that the measured results show an agreement with the simulated ones especially for the measured and simulated magnitude distributions. However, in Figure 4b it is still difficult to clearly observe what topological charges are encoded in the measured phase. To reveal, three closed contours with different radii are defined in the phase distribution of Figure 4b, corresponding to black, purple, and magenta solid circles denoted by i, ii, and iii, respectively. Along the contours, the measured and simulated phase distributions are compared, as illustrated in Figure 4b(i–iii), from which it can be observed that although the measured phase distributions are slightly distorted, it is still in accordance with the simulated ones. Therefore, it can be confirmed that the measured plasmonic vortex in spin‐+ 1 state carries multiple topological charges of −2, +6, and −10, as the simulated one does. The discrepancy between the measured results and the simulated ones primarily results from two aspects: First, the in situ observations rely on the direct measurements of SPP near fields by a probe scanning the sample surface. However, the exponential decay feature of SPPs requires that the probe has to be placed to the sample surface very close, which increases the difficulty of controlling the probe to detect the field distributions on a fixed *z* plane. The probe is frequently damaged. This issue is further amplified by the surface fabrication quality of the sample. Second, for the deuterogenic plasmonic vortices vortex modes with orders of −2 and +6, their field amplitudes are intrinsically very weak [11]. They contribute only a small fraction of the total near‐field signal compared with the dominant vortex with the order of −10, which makes them particularly difficult to resolve clearly in a direct near‐field scan. To further verify the influence of the signal‐arm optical intensity on the heterodyne detection, a control experiment without the sample was carried out. The measured amplitude of the heterodyne signal decreases proportionally with the signal‐arm optical intensity (see Figure S12 in Section S8). The plasmonic scalar vortex in spin‐−1 state excited by | −, 0〉 is measured as well, which also well demonstrates our simulated results in the right column in Figure 2a (see Figure S13 in Section S8). The measured polarization vortex and the auxiliary complex scalar field Φ in the transverse SPP fields are visualized in Figure 4c, corresponding to the simulated results in Figure 3c(i–iii). Although the measured results are slightly distorted, they still show good similarity to the simulated ones.

## 5 Discussions

Apart from the fundamental and vectorial selection rules for exciting phase and polarization singularities, our group‐theoretical approach can be further applied to reveal the selection rule for exciting and engineering higher‐dimensional plasmonic singularities, e.g., 3D singularities like skyrmions, merons, and their transformations or extensions [39]. In singular optics, higher‐dimensional singularities (e.g., skyrmions, merons, hopfions, knots, links, and Möbius strips)) can be understood from the perspective of lower‐dimensional singularities [2]. For example, a field skyrmion in SPPs can be regarded as the synthesis of a trivial scalar vortex with no phase singularity and a polarization vortex with polarization singularity in the out‐of‐ and in‐plane SPP fields [2]. Therefore, we can take the fundamental and vectorial rules to build up selection rules for exciting higher‐dimensional plasmonic singularities. Due to the length restrictions of this work, here we only investigate the excitation of a 3D plasmonic singularity, i.e., topological quasiparticles, including skyrmions and merons (half skyrmions), as well as their transformations and extensions (see Methods). Our primary finding here is that the identity irrep (irrep index *j*  =  0) of the underlying group governs the excitation of the topological quasiparticles in SPPs. Namely, our considered topological quasiparticles belong to the identity irrep of the *C_M_* group and to excite them, the incident light should exhibit the same symmetry of the irrep or carry the total angular momenta of *Mq*ℏ according to Equation (2), i.e.,

(5) l+σ=Mq

This equation serves as a selection rule for exciting our considered topological quasiparticles in SPPs. To demonstrate this, we provide an example in Methods, presenting some interesting phenomena. (1) The incident light | +, −1〉 can excite a nest of merons consisting of a Bloch‐type meron at the central region and a circular meron lattice (a ring‐like lattice consisting of staggered bubble‐type merons and antimerons) at the outer annular region. And (2) increasing the incident light orbit can realize the transitions of the meron nest: subsequently using the incident light from | +, −1〉, via | +, −9〉, to | +, −17〉 to excite the PVL, the quasiparticle configuration will be transformed from the meron nest, via Néel‐type skyrmion, finally to a bubble‐type skyrmion. This is a purely optical‐control approach to tune the configurations of topological quasiparticles, different from our previous finding by changing the electric size of photonic microstructures [33].

To show the potential power of our derived selection rules in study various singularity‐related phenomena, we further apply them to analyze a recent emergent phenomena of plasmonic quasicrystals, an ideal platform to study topological physics in higher dimensions [16]. The scalar and even vectorial field quasi‐lattice patterns can be understood within the framework of our generalized selection rules (see an example of a plasmonic system with *D*_5_ group symmetries in Methods). More examples of plasmonic systems with *C*_2_ and *C*_3_ group symmetries and a microwave plasmonic system with *D*_8_ group symmetries further demonstrate the broad applicability of our group‐theoretical approach (see Figures S14 and S15 in Section S9 and Figures S16 and S17 in Section S10).

In addition, it is worth noting that our proposed selection rules also imply the peculiar rest mass of SPPs as a bosonic quasiparticle in the context of quantum electrodynamics [28]. It is known that a photon in vacuum is a gauge boson, indicating that the photon in vacuum can take only two spin states, i.e., spin‐±1 states, and its spin‐0 state is forbidden [40]. There is an interchangeable statement that a photon in vacuum is a gauge boson, i.e., a photon in vacuum has no rest mass. The spin‐0 state in our proposed rules suggest that the SPPs as bosonic quasiparticles created in light‐matter interactions are quite different from the photon and should own rest mass. The massive SPPs have been investigated very recently and show great potential in nonlinear optics [41], further reflecting the validity and potential of our obtained selection rules.

Finally, our symmetry‐imposed selection rules can serve as a general principle to study the symmetry‐ and singularity‐related phenomena in diverse wave systems, e.g., polarization singularities in momentum space in photonic systems [42], skyrmions in acoustic systems [43], or exceptional points in spin wave systems [44].

## 6 Methods

### 6.1 Experimental Setup for Near‐Field Measurements

To visualize the plasmonic vortices, the spin‐selective and phase‐resolved scanning near‐field optical microscope (SSPR‐SNOM) was established. The incident beam from a HeNe laser (Pacific Lasertec, 25‐LHP‐928, 633 nm, continuous wave) was directed through a custom‐designed optical path to excite the sample. Heterodyne interferometry was employed to achieve a light frequency difference (∆f) of 20 kHz between the signal and reference branches, which was achieved by utilizing acousto‐optic modulators (IntraAction, AOM_402AF1). Circularly polarized beams were generated in both branches using a polarization compensation unit. In the signal optical path, near‐field vortices were detected with nanoscale resolution using an aperture‐type probe on a commercial scanning near‐field optical microscope platform (NT‐MDT, SNLG112NTF). By selectively adjusting the spin state in the reference path, the interference profile was captured using a photomultiplier tube (Hamamatsu, H10723‐20) based on a specific polarized basis. Amplitude and phase distributions were demodulated simultaneously using a lock‐in amplifier (LIA). The corresponding left and right circular components were analyzed, respectively.

### 6.2 3D Plasmonic Singularities: Plasmonic Topological Quasiparticles

Topological quasiparticles in SPPs can be constructed by different field vectors of SPPs, e.g., electric/magnetic‐field vectors [1, 45], local spin vectors [5], and Stokes vectors(pseudo‐spin vectors) [39] etc. Here, we focus on the topological quasiparticles synthesized with plasmonic electric‐field vectors. First, we use the LG mode | +, −1〉 illuminating the PVL. In this case, the SPP field belonging to the identity irrep (i.e., irrep index *j*  =  0) is excited according to Equation (2) (see Extended Data Figure 1). From Extended Data Figure 1a, it can be observed that an isolated Bloch‐type meron is formed at the central region enclosed by the white or black dashed circle (also see zoomed‐in plot in Extended Data Figure 1b); and a circular lattice consisting of bubble‐type merons and antimerons is formed in the annular region enclosed by the dashed and solid circles. The central meron and the outer circular meron lattice form a nest of merons. The topological invariant/charge of the central meron in Extended Data Figure 1b can be characterized by the skyrmion number of 1/2 evaluated by,

(6) $$N=\frac{1}{4\pi }\int \;\;\;\int_{A}e\cdot \left(\frac{\partial e}{\partial x}\times \frac{\partial e}{\partial y}\right)dxdy$$

In the above, **e**  =  Re{*E_x_*,*E_y_*,*E_z_*}/|**E**| is the normalized electric field. It is worth noting that the average skyrmion number of the meron is 0 within one optical cycle as reported previously, which might be useful in ultrafast optics [1]. The excited plasmonic vortices are illustrated in Sections S4 and S5. The boundary of the central meron is defined by the L‐line singularity map in the polarization vortex in the insets denoted by *j*  =  0 of Figure S8. The circular meron lattice in the outer annular region consists of staggered bubble‐type merons and antimerons. Then, we use another LG modes to excite the identity irrep according to Equation (2), e.g., | +, −9〉 and | +, −17〉. Extended Data Figure 2a shows the excited SPP field by | +, −9〉, from which a Néel‐type skyrmion with a skyrmion number of 1 can be observed instead of the meron nest in the case of | +, −1〉. Extended Data Figure 2b shows the excited SPP field by | +, −9〉, from which a bubble‐type skyrmion with a skyrmion number of 1 can be observed, i.e., an optical domain wall is formed in the vectorial configuration [1, 33]. From the above discussions, we find an interesting phenomenon that by changing the orbit of incident light from −1, via −9, to −17, we realize the transitions of the topological quasiparticles in SPPs from meron nest, via Néel‐type skyrmion, to bubble‐type skyrmion, even though the same irrep (i.e., identity irrep) is excited by the three incident fields.

**Extended Data** Figure 1. Meron field configuration excited by | +, −1〉 in the identity irrep (i.e., the ***A***_1_ irrep): (a) Multiplexed meron configuration consisting of an isolated meron at the central region enclosed by the dashed circle and a circular meron lattice at the annular region enclosed by the dashed and solid circles; (b) the zoomed‐in vector distribution of the isolated meron at the central region in (a). In (a) and (b), ***R***_***o***_ = * *1.95 ***λ***_***spp***_ and ***R***_***i***_ = * *0.46 ***λ***_***spp***_. The insets at the bottom show the intensity distributions of the SPP fields, and the colors of the vectors are coded from blue to red to denote the ratio of ***e***_***z***_/|**e**| varying from ‐1 to 1.


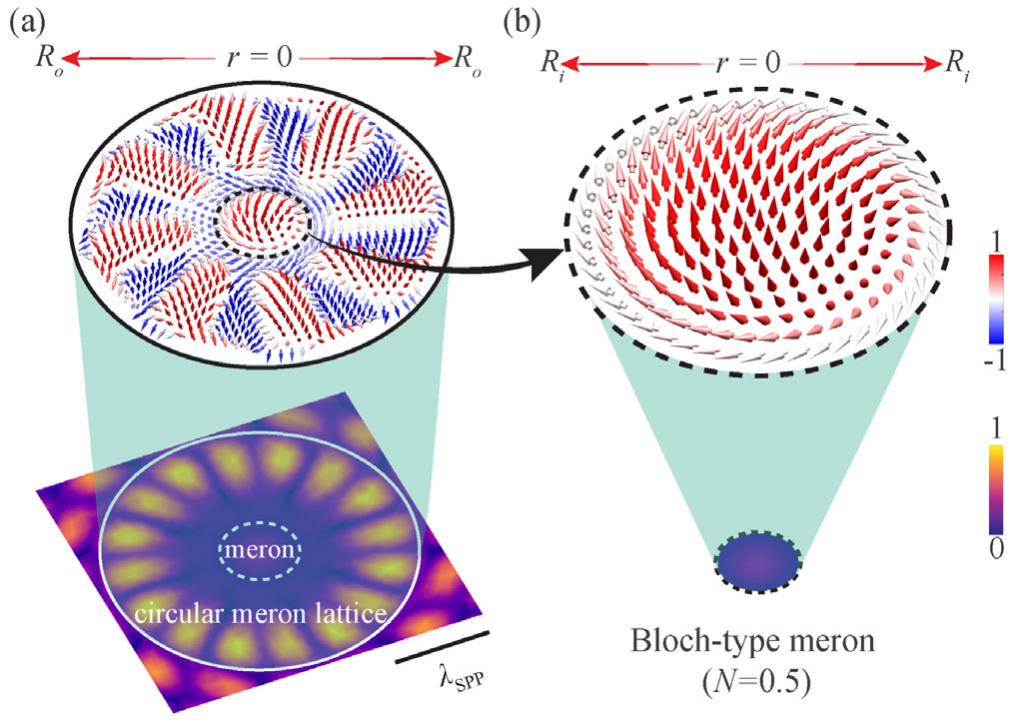


**Extended Data** Figure 2. Transformation between Néel‐ and bubble‐type skyrmions in the identity irrep: (a) Néel‐type skyrmion excited by | +, −9〉; (a) Bubble‐type skyrmion excited by | +, −17〉. In (a) and (b), *R*_1_ =  0.59λ_*spp*_ and *R*_2_ =  0.73λ_*spp*_. The bottom inset shows the intensity distributions of the SPP fields, and the colors of the vectors are coded from blue to red to denote the ratio of *e_z_*/|**e**| varying from ‐1 to 1. In (b), a so‐called optical domain wall can be observed which segregates the vector‐up and ‐down regions.


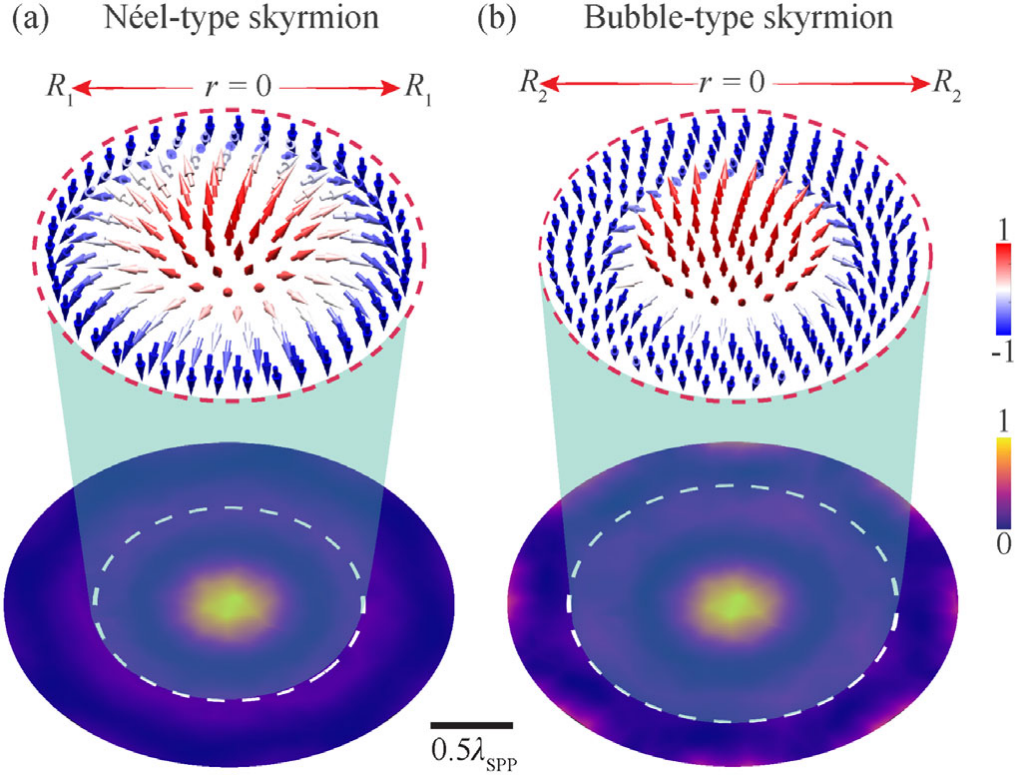


### 6.3 Multidimensional Singularities in Plasmonic Quasicrystals

To demonstrate the broad applicability of our derived selection rules, here we investigate an example related to the very recently reported plasmonic quasicrystal phenomenon [16]. The plasmonic quasicrystals can be constructed by polygonal PVLs which hold discrete rotational symmetries except for two‐, three‐, four‐, and six‐fold ones. Here we take the five‐fold rotational symmetry as an example just like the reported case (see the pentagonal PVL in Extended Data Figure 3a) [16]. The symmetries of the pentagonal PVL form a *D*_5_ group, which is the direct product of *C*_5_ group and *D*_1_ group, i.e., *D*_5_ = *C*_5_ ⊗*D*_1_, where *D*_1_ group is the reflection group isomorphic to *C*_2_ group. Therefore, irreps of the *D*_5_ group can be labeled by the irrep index of the *C*_5_ group. The *D*_5_ group has two 1D irreps (marked with *A*_1_ and *A*_2_) and two 2D irreps (marked with *E*_1_ and *E*_2_). Theoretically, there are six quasi‐lattice patterns mutually orthogonal, i.e., two nondegenerate patterns belonging to the *A*_1_ and *A*_2_ irreps and two pairs of doubly degenerate patterns belonging to the *E*_1_ and *E*_2_ irreps (see Table S2 in Section S11).

**Extended Data** Figure 3. Plasmonic quasicrystal: (a) The LG mode | − 1, 4〉 at the wavelength of ***λ *** = * *750 *** nm*** shines on the PVL with the ***D***_5_ group symmetries, where ***w *** = * *7.4 ***λ***_**spp**_, ***λ***_**spp**_ = * *726 *** nm***, and the width of the each slit is ***λ***_**spp**_/2; (b) The deuterogenic scalar vortices in three spin states and the corresponding topological charges; (c) The polarization vortex in the transverse plasmonic field carrying topological charge vector (***I***_***o***_, ***I***_***e***_)* * = (− 3/2, 1/2)* *; and (d) the normalized Stokes vector pattern, where a higher‐order Stokes meron with skyrmion number of ***N *** = * *3/2 appears at the central region enclosed by the white dashed Pentagon in (c). The boundary of the Stokes meron, i.e., the white dashed Pentagon in (c), is defined by the L‐line singularity map where the transverse polarization ellipses are always linear. In (e), the colors of the vectors are coded from blue to red to denote the third component of the normalized Stokes vectors varying from −1 to 1.


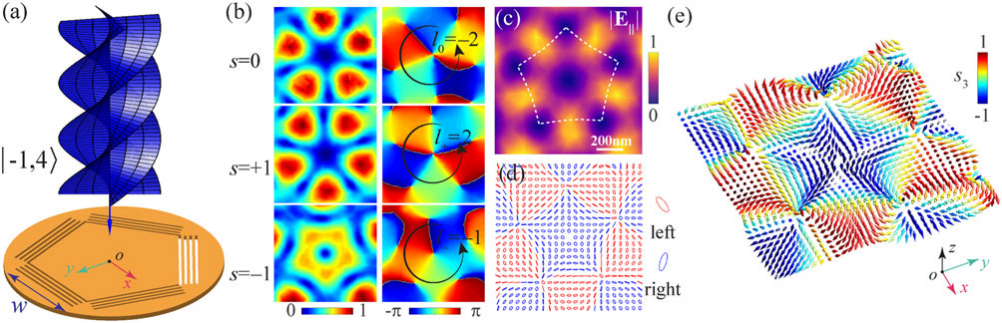


Different patterns of plasmonic quasi‐lattices can be created in the PVL by choosing the incident light with proper spins and orbits. Here we choose the incident light as an LG mode with spin and orbit numbers of −1 and 4, i.e., | − 1, 4〉 (see Extended Data Figure 3a). According to Equation (2), the quasi‐lattice pattern belonging to the first dimension of the *E*_2_ irrep indexed by *j*  =  2 will be excited. The excited SPP fields are shown in Extended Data Figure 3b–d, where only the central patterns are depicted to simplify the analysis and validate our generalized selection rules. From Extended Data Figure 3b, it can be observed that three plasmonic scalar vortices are formed in three plasmonic spin states, whose topological charges are *l*_0_ =   − 2,  *l*_+_ =  2, and *l*_−_ =   − 1, agreeing well with our selection rule in Equation (3) by instituting *M*  =  5 into the equation. Further, we analyze the vector polarization singularity in the transverse SPP fields, as shown in Extended Data Figure 3c,d, from which it can be observed that at the central region enclosed by the white dashed pentagon, a dark C‐point polarization singularity with right handedness occurs. The topological charge vector of the polarization singularity is **I**  = (*I_o_*,*I_e_*)  = (− 3/2, 1/2) . As an extension of our discussion to topological quasiparticles, we further plot the normalized Stokes vector patterns of the polarization morphology in Extended Data Figure 3d (see definition of Stokes vector in Section S5), as shown in Extended Data Figure 3e, from which it can be observed that the normalized Stokes vector pattern actually exhibit a topological configuration of a higher‐order topological meron whose boundary is defined by the L‐line singularity map where the polarization ellipses are linear. The skyrmion number of the meron can be evaluated as *N*  =  *p* · *m*  =  2*I_o_* · *m*  = 3/2 , where *p* and *m* denote polarity and vorticity of the meron and in our case, *m*  =   − 1/2 and *p*  =  2 *I_o_* =   − 3 [32]. The quasi‐lattice patterns in the larger region beyond the central one can be analyzed by a similar way, i.e., the patterns can be understood within the framework of nested plasmonic singularity superposition. Topological charges of the nested plasmonic singularities are strictly governed by our proposed generalized selection rules.

## Author Contributions

**Yuanjie Yang**: methodology, formal analysis, Writing – review and editing. **Fei Han**: validation, investigation, resources, writing – review and editing. **Anxue Zhang**: methodology, writing – review and editing, supervision, formal analysis. **Zhongwei Jin**: data curation, investigation, visualization, writing – review and editing, resources. **Ewald Janssens**: supervision, resources, writing – review and editing. **Niels Verellen**: resources, validation, investigation, writing – review and editing. **Weijin Chen**: formal analysis, investigation, data curation, writing – review and editing. **Pengyi Feng**: methodology, software, data curation, validation, formal analysis, visualization, writing – review and editing. **Jie Yang**: conceptualization, funding acquisition, writing – original draft, methodology, writing – review and editing, formal analysis, visualization. **Jiafu Wang**: methodology, formal analysis, supervision, funding acquisition, writing – review and editing, project administration. **Benfeng Bai**: validation, formal analysis, project administration, funding acquisition, writing – review and editing. **Cheng‐Wei Qiu**: writing – review and editing, project administration, funding acquisition, supervision, formal analysis. **Jincheng Ni**: investigation, data curation, formal analysis.

## Conflicts of Interest

The authors declare no conflicts of interest.

## Supporting information




**Supporting File**: advs77043‐sup‐0001‐SuppMat.docx.

## Data Availability

The data that support the findings of this study are available from the corresponding author upon reasonable request.
