# Supplementary material for: Symmetry‐Imposed Selection Rules for Excitations of Nontrivial Plasmonic Topologies
Source: Adv Sci (Weinh). 2026 Aug 10;13(62):e77043. doi: 10.1002/advs.77043 (PMC13455830; doi:10.1002/advs.77043)
Supplement: Supplementary file 1 [file ADVS-13-e77043-s001.docx]

Supporting Information for

Symmetry-imposed selection rules for excitations of nontrivial plasmonic topologies

Jie Yang^1,2,3†^, Pengyi Feng^4†^, Fei Han^5,6^, Weijin Chen^7^, Zhongwei Jin*‬‬‬‬*^8^, Jincheng Ni^7^, Yuanjie Yang^9^, Anxue Zhang^10^, Guy A E Vandenbosch^3^, Niels Verellen^6,5^, Ewald Janssens^5^, Jiafu Wang^1,10,11^ *, Benfeng Bai^4^*, Cheng-Wei Qiu^7^*,‬‬‬‬‬‬‬‬‬‬‬‬‬‬‬‬‬‬‬‬‬‬‬‬‬‬‬‬‬‬‬‬‬‬‬‬‬‬‬‬‬‬‬‬‬‬‬‬‬‬‬‬‬‬‬‬‬‬ and Xuezhi Zheng^12,3^**‬‬‬‬‬‬‬‬‬‬‬‬‬‬‬‬‬‬‬‬‬‬‬‬‬‬‬‬‬‬‬‬‬‬‬‬‬‬‬‬‬‬‬‬‬‬‬‬‬‬‬‬‬‬‬‬‬‬‬‬‬‬‬‬‬‬‬‬‬‬‬‬*

Contents

[SI 1. Symmetry-classified eigen plasmonic vortices	2](#_Toc235198515)

[SI 1.1. Volume integral equation formulism for light-matter interaction	2](#_Toc235198516)

[SI 1.2. Symmetries	4](#_Toc235198517)

[SI 1.3. Commutative relation and symmetry matching condition	5](#_Toc235198518)

[SI 2. Derivation of the generalized selection rules	7](#_Toc235198519)

[SI 3. Derivation of the selection rules for the case of U(1) group symmetries	10](#_Toc235198520)

[SI 4. The excited plasmonic scalar vortices in three spin states of SPPs	12](#_Toc235198521)

[SI 5. The excited plasmonic polarization vortices	16](#_Toc235198522)

[SI 6. Revisiting dynamic topological charges of plasmonic polarization vortices in cylindrical bases	23](#_Toc235198523)

[SI 7. Sample fabrications	25](#_Toc235198524)

[SI 8. Experimental setup and verifications	27](#_Toc235198525)

[SI 9. Demonstrations in the cases of C2 and C3 group symmetries	31](#_Toc235198526)

[SI 10. Demonstration in microwave plasmonic structure	34](#_Toc235198527)

[SI 11. Symmetries of pentagonal plasmonic quasicrystals	37](#_Toc235198528)

# SI 1. Symmetry-classified eigen plasmonic vortices

## SI 1.1. Volume integral equation formulism for light-matter interaction

Fig. S1 illustration of vortex light-PVL interaction. In the figure, some examples of the plasmonic/photonic microstructures producing multidimensional vortices in the process of plasmonic/photonic SOI are listed: i^1,2^, ii^3–5^, iii^6–8^, iv^9^, v^10,11^, and vi^12,13^. Our obtained selection rules apply to these microstructures as well.

The interaction between incident light and the plasmonic vortex lens (PVLs) ) (see Fig. S1) can be described by a volume integral equation^14–17^, which can be formulated as below,

$$\hat{Z}\left\{\mathbf{J}\left(\mathbf{r},\omega\right)\right\}=\mathbf{E}_{inc}\left(\mathbf{r},\omega\right).$$

In above equation, $\hat{Z}$ denotes an impedance operator that links the incident field $\mathbf{E}_{inc}\left( \mathbf{r} \right)$ with the induced current **J**(**r**,*ꞷ*) flowing in the PVL. The above operator equation can be derived from the following procedures. In the main text, the above operator is written as $\hat{Z}|\left. J \right\rangle=|\left. \sigma\mathbf{,}l \right\rangle$ in Dirac notation. To obtain the expression of the impedance operator, we start from the field relation that the total field in the space is composed of the incident and the scattered fields, i.e.,

$$\mathbf{E}_{tot}\left(\mathbf{r}\right)=\mathbf{E}_{inc}\left(\mathbf{r}\right)+\mathbf{E}_{scat}\left(\mathbf{r}\right).$$

The total field can be linked with the induced current via the volume equivalence principle^14–17^,

$$\mathbf{E}_{tot}\left(\mathbf{r}\right)=-\frac{\mathbf{J}\left(\mathbf{r}\right)}{i\omega\left(\varepsilon_{V}-\varepsilon\right)}.$$

In above equation, $\varepsilon_{V}$ is the permittivity of the material filling the PVL, which is dependent on spatial positions and frequencies; $\varepsilon$ is the permittivity of the surrounding material. The scattered field is generated by the induced current and can be written as,

$$\mathbf{E}_{scat}\left(\mathbf{r}\right)=i\omega\mu\int_{V} \overline{\mathbf{G}}\left(\mathbf{r},\mathbf{r}'\right)\cdot\mathbf{J}\left(\mathbf{r}'\right)d\mathbf{r}'.$$

In above equation, $\bar{\mathbf{G}}$ is the dyadic Green’s function and $\mu$ is the permeability of the surrounding material. $\mathbf{r}'$and$\mathbf{r}$ denote the source and observation points, respectively.

Use Eqs. and in Eq. . We can obtain the following operator equation,

$$-\frac{\mathbf{J}\left(\mathbf{r}\right)}{i\omega\left(\varepsilon_{V}-\varepsilon\right)}-i\omega\mu\int_{V} \overline{\mathbf{G}}\left(\mathbf{r},\mathbf{r}'\right)\cdot\mathbf{J}\left(\mathbf{r}'\right)d\mathbf{r}'=\mathbf{E}_{\mathbf{inc}}\left(\mathbf{r}\right).$$

The above equation is the volume integral equation and can be written in an operator form, with the impedance operator $\hat{Z}$ in Eq. defined as

$$\hat{Z}\left\{\mathbf{J}\left(\mathbf{r}\right)\right\}=-\frac{\mathbf{J}\left(\mathbf{r}\right)}{i\omega\left(\varepsilon_{V}-\varepsilon\right)}-i\omega\mu\int_{V} \overline{\mathbf{G}}\left(\mathbf{r},\mathbf{r}'\right)\cdot\mathbf{J}\left(\mathbf{r}'\right)d\mathbf{r}'.$$

Associated with Eq. , an eigenvalue problem of the $Z$ operator is defined as

$$\hat{Z}\left\{\mathbf{j}\left(\mathbf{r},\omega\right)\right\}=\lambda\left(\omega\right)\mathbf{j}\left(\mathbf{r},\omega\right).$$

By Eq. , we can conveniently study the electromagnetic (EM) properties of the PVL which are independent from the incident field.

Note that Eqs. - are applicable to describe the interaction of light and the microstructures made of metals operating at optical, since optical metals behave like lossy dielectrics. The equations also applies when microstructures are made of semiconductors merely considering that the $\varepsilon_{V}$ take a negative imaginary part since the gain in semiconductors. When the microstructures are made of metals and operate at lower frequencies like far-infrared, terahertz or microwaves, the interaction is described by a surface integral equation,

$$-\mathbf{n}\left(\mathbf{r}\right)\times\text{i}\omega\mu_{0}\int_{S} \overline{\mathbf{G}}\left(\mathbf{r,}\mathbf{r}'\right)\times\mathbf{J}\left(\mathbf{r}'\right)\mathbf{d}\mathbf{r}'=\mathbf{n}\left(\mathbf{r}\right)\times\mathbf{E}_{\mathbf{inc}}\left(\mathbf{r}\right)$$

since the metal can be regarded as perfect electric conductor (PEC) in these frequency bands. In the above, $S$ denotes the surface of the metallic microstructures and $\mathbf{n}$ denotes the unit normal vector of the surface. The above can also be written as the form of Eqs. -, as below,

$$\hat{Z}\left\{\mathbf{J}\left(\mathbf{r}'\right)\right\}=\mathbf{V}_{\mathbf{inc}}\left(\mathbf{r}\right)\Rightarrow\left\{\begin{matrix} \hat{Z}=-\mathbf{n}\left(\mathbf{r}\right)\times\text{i}\omega\mu_{0}\int_{S} \overline{\mathbf{G}}\left(\mathbf{r,}\mathbf{r}'\right)\mathbf{\times d}\mathbf{r}' \\ \mathbf{V}_{\mathbf{inc}}\left(\mathbf{r}\right)=\mathbf{n}\left(\mathbf{r}\right)\times\mathbf{E}_{\mathbf{inc}}\left(\mathbf{r}\right) \end{matrix}\right..$$

## SI 1.2. Symmetries

The *M*-fold rotational symmetries held by the PVL form a cyclic group, i.e., the $C_{M}$ group. The irreducible representations (irreps) of the $C_{M}$ group is presented in Table S1, where $C_{M}^{m}$ denotes a ${2m\pi}/M$ rotation, $\Gamma_{j}$ denotes the *j*^th^ irrep. As an abelian group, all irreps of the $C_{M}$ group are one-dimensional and the number of the irreps equals the number of symmetry operations^18^.

**Table S1** The irreps of $C_{M}$ group, where $\varepsilon=e^{{i2\pi}/M}$. Replacing $M=8$ in the table we obtain the irreps of $C_{8}$ group.

| $\boldsymbol{C}_{\boldsymbol{M}}$ | $C_{M}^{m}$ |
| --- | --- |
| $\boldsymbol{\Gamma}_{\boldsymbol{j}}$ | $\left( \varepsilon^{j} \right)^{m}$ |

For the *j*^th^ irrep, one can define the so-called projection operator as below^19^,

$$\hat{P}^{j}=\frac{1}{M}\sum_{R} \left(\chi_{R}^{j}\right)^{*}\hat{P}_{R}.$$

In the definition, $R$ represents a rotation ($R\in\left\{E,C_{M},C_{M}^{2},...C_{M}^{m},...C_{M}^{M-1}\right\}$, *m*=0,1,…,(*M*-1)); $\hat{P}_{R}$ is a transformation operator which applies a symmetry operation $R$ to an arbitrary function; $\chi_{R}^{j}$ is the *j*^th^ irrep of a rotation $R$ which can be obtained from the Table S1. The asterisk denotes complex conjugation. Applying the projection operator to an arbitrary function leads to a projected function, i.e., $\hat{P}^{j}\mathbf{f}\left(\mathbf{r}\right)=\mathbf{f}^{j}\left(\mathbf{r}\right)$ , where $\mathbf{f}$ can denote either a vector function or a scalar function. The projected function is also the eigenfunction of the projection operator and the related eigenvalue is +1, i.e.,$\hat{P}^{j}\mathbf{f}^{j}\left(\mathbf{r}\right)=\mathbf{f}^{j}\left(\mathbf{r}\right)$. Please see more detailed discussions about the character table and projection operators of $C_{M}$ group in the pedagogical textbook^19^.

## SI 1.3. Commutative relation and symmetry matching condition

Here, we consider the interplay between the symmetries and the light-PVL interaction. It can be proven that each projection operator of the $C_{M}$ group and the $\hat{Z}$ operator is commutative^15–17^, i.e.,$\hat{P}^{j}\hat{Z}=\hat{Z}\hat{P}^{j}$. Due to the commutative relation, the projection operators and the $Z$ operator share the same set of eigenfuncitons, that is,

$$\hat{Z}\left\{\mathbf{j}^{j}\left(\mathbf{r},\omega\right)\right\}=\lambda^{j}\left(\omega\right)\mathbf{j}^{j}\left(\mathbf{r},\omega\right),\text{ }\hat{P}^{j}\mathbf{j}^{j}\left(\mathbf{r},\omega\right)=\mathbf{j}^{j}\left(\mathbf{r},\omega\right).$$

The first formula in Eq. suggests that within the context of the commutative relation, the eigenfunctions of the projection operators can be physically interpreted as “eigencurrents” supported by the PVL. Due to the commutative relation between each projection operator and the impedance operator, the Hilbert space spanned by the eigenfunctions of the impedance operator $\hat{Z}$ can be divided into *M* one-dimensional subspaces, which correspond to the *M* irreps of the $C_{M}$ group. As a result, there are only *M* possible categories of the eigen current distributions that can be supported by the PVL, and, accordingly, *M* possible categories of the electric field distributions that can be radiated (or scattered) by the PVL. Therefore, for the PVL with $C_{8}$ group symmetry, there are only $8$ possible categories of electric field distributions, i.e., eigenmodes. Following conclusions in our previous works^20,21^, for a certain category of eigenmodes its three components with different polarization states exhibit scalar vortex modes carrying different topological charges evaluating as Eq. (1) in the main text.

Since the eigenfunctions of the projection operator which can be derived purely based on the symmetry arguments (i.e., solving the eigenvalue problem of the projection operators), their radiated fields reflect the most fundamental restrictions laid down by the symmetries of the structure on its scattered fields.

Again, due to the commutative relation, applying the projection operator to Eq. which describes the process of the light-PVL interaction, leads to

$$\hat{Z}\left\{\mathbf{j}^{j}\left(\mathbf{r},\omega\right)\right\}=\hat{P}^{j}\mathbf{E}_{inc}\left(\mathbf{r},\omega\right),\text{ }\hat{P}^{j}\mathbf{E}_{inc}\left(\mathbf{r},\omega\right)\neq0.$$

Eq. suggests that, to excite the eigencurrents $\mathbf{j}^{j}$ belonging to the *j*^th^ irrep, a given incident field must have a non-vanishing projection along the same irrep, that is, right hand of side in above equation must not vanish. In this sense, the symmetry of incident field matches with the irrep. In a special case, if the incident field is invariant under the transformation of a projection operator, i.e.,

$$\hat{P}^{j}\mathbf{E}_{inc}\left(\mathbf{r},\omega\right)=\mathbf{E}_{inc}\left(\mathbf{r},\omega\right).$$

The eigencurrents belonging to the same irrep will be exclusively induced by the incident field.

# SI 2. Derivation of the generalized selection rules

The PVLs with rotational symmetries are normally excited by a circularly polarized vortex beam $|\left. \sigma,l \right\rangle$, whose expression can be written as,

In above equation,  denotes matrix transposition. The operator equation of the PVL can be derived by substituting the indent electric field into Eq.

$$\hat{Z}(\mathbf{J}(\mathbf{r}'))=|\sigma,l\rangle.$$

Applying the projection operator of the *j*^th^ irrep on both sides of Eq. and further exploiting the commutative relation between the projection operator and the impedance operator, we can obtain,

$$\hat{Z}\left(\hat{P}^{j}(\mathbf{J}(\mathbf{r}'))\right)=\hat{P}^{j}|\sigma,l\rangle.$$

The above equation implies that the eigen current of the *j*^th^ irrep is excited by the incident light if $\hat{P}^{j}|\sigma,l\rangle\neq0$. Specifically, the incident field is invariant under transformation of a projection operator, i.e.,

$$\hat{P}^{j}|\sigma,l\rangle=|\sigma,l\rangle.$$

Once the above condition satisfied, the *j*^th^ irrep will be exclusively excited by the vortex beam$|\sigma,l\rangle$. Next, we want to know which irrep is excited by the incident electric field with spin and orbit quantum numbers *σ* and *l*,

$$\hat{P}^{j}|\sigma,l\rangle=\frac{1}{M}\sum_{R} \left(e^{imj\frac{2\pi}{M}}\right)^{*}\hat{P}_{R}|\sigma,l\rangle.$$

Consider the following term,

Firstly, we re-write the term of as,

where *ρ* and *φ* are the radius and the azimuthal angle of the cylindrical coordinate system, respectively. Use Eq. in Eq. ,

$$\hat{P}_{R}|\sigma,l\rangle=\bar{\mathbf{R}}\cdot(\hat{\mathbf{x}}+i\sigma\hat{\mathbf{y}})\left(\frac{\mathbf{d}}{\rho}\cdot\bar{\mathbf{R}}^{-1}\mathbf{r}\right)^{l}E_{0}e^{-i(\omega t-\mathbf{k}\cdot\bar{\mathbf{R}}^{-1}\mathbf{r})}.$$

where $\bar{\mathbf{R}}$ is the transformation matrix of the rotation operation *R* and is given as below.

$$\bar{\mathbf{R}}=\left[\begin{matrix} \cos\theta& -\sin\theta& 0 \\ \sin\theta& \cos\theta& 0 \\ 0 & 0 & 1 \end{matrix}\right],\text{ }\theta=m\frac{2\pi}{M}.$$

After some derivations, we can obtain following three identities,

$$\bar{\mathbf{R}}\cdot(\hat{\mathbf{x}}+i\sigma\hat{\mathbf{y}})=\left[\begin{matrix} \cos\theta& -\sin\theta& 0 \\ \sin\theta& \cos\theta& 0 \\ 0 & 0 & 1 \end{matrix}\right]\cdot\left[\begin{matrix} 1 \\ i\sigma\\ 0 \end{matrix}\right]=\left[\begin{matrix} 1 \\ i\sigma\\ 0 \end{matrix}\right]e^{-i\sigma\theta},$$

$$\bar{\mathbf{R}}^{-1}\mathbf{r}=\left[\begin{matrix} \cos\theta& \sin\theta& 0 \\ -\sin\theta& \cos\theta& 0 \\ 0 & 0 & 1 \end{matrix}\right]\cdot\left[\begin{matrix} x \\ y \\ z \end{matrix}\right]=\left[\begin{matrix} x\cos\theta+y\sin\theta\\ -x\sin\theta+y\cos\theta\\ z \end{matrix}\right],\text{ }$$

Use Eq.s - in Eq. ,

Use Eq. in Eq. ,

In Eq. , we see the sum of a geometric progress. By using the following identity,

$$\sum_{m=0}^{M-1} e^{-i(j+l+\sigma)m\frac{2\pi}{M}}=\left\{\begin{matrix} M,\text{ }j+l+\sigma=Mq \\ 0,\text{ }j+l+\sigma\neq Mq \end{matrix}\right.,\text{ }q=0,\pm1,\pm2,..$$

we can obtain,

$$\hat{P}^{j}|\sigma,l\rangle=\left\{\begin{matrix} 0,\text{ }j+l+\sigma\neq Mq \\ |\sigma,l\rangle,\text{ }j+l+\sigma=Mq \end{matrix}\right..$$

Therefore, we conclude that the *j*^th^ irrep will be excited by $|\sigma,l\rangle$if and only if the following equation is satisfied,

$$j+l+\sigma=Mq,q=0,\pm1,\pm2,...$$

# SI 3. Derivation of the selection rules for the case of U(1) group symmetries

Here, we consider a PVL lying on the $xy$ plane is invariant under the continuous rotation with respect to the $z$ axis, i.e., the PVL holds SO(2) or U(1) group symmetries. A strategy similar to the case of discrete rotational symmetries is adopted. Divide a full rotation 2*π* into $M$ rotations, each of which rotates the PVL by a geometric angle of $\Delta\theta=\frac{2\pi}{M}$. Then, we can construct the projection operator for the *j*^th^ irrep by taking the limit of a sum as$M\to+\infty$,

$$\hat{P}^{j}=\lim_{M\to+\infty} \frac{1}{M}\sum_{R} \left(\chi_{R}^{j}\right)^{*}\hat{P}_{R}.$$

In this equation, $R$ represents a geometric rotation ($R\in\left\{E,C_{M},C_{M}^{2},...C_{M}^{m},...C_{M}^{M-1}\right\}$, *m*=0,1,…*M*-1 and $C_{M}^{m}$ is the $\Delta\theta={2m\pi}/M$ rotational operation with respect to the *z* axis) which leaves the PVL invariant; $\chi_{R}^{j}$ is the *j*^th^ irrep of the rotation operation $R$ and $\chi_{R}^{j}=e^{ijm\Delta\theta}$;and $\hat{P}_{R}$ represents the transformation operator corresponding to the geometric rotation $R$. With the similar procedures to the discrete case, we apply the projection operator to the incident circularly polarized vortex beam$|\sigma,l\rangle$. After some algebraic manipulations, we obtain,

By using the following identity,

$$\int_{0}^{2\pi} e^{-i(j+l+\sigma)\theta}d\theta=\left\{\begin{matrix} 2\pi,\text{ }j+l+\sigma=0 \\ 0,\text{ }j+l+\sigma\neq0 \end{matrix}\right..$$

we can obtain,

$$\hat{P}^{j}|\sigma,l\rangle=\left\{\begin{matrix} 0,\text{ }j+l+\sigma\neq0 \\ |\sigma,l\rangle,\text{ }j+l+\sigma=0 \end{matrix}\right..$$

By further using the relation between *j* and *l_s_* in this case, i.e., *l_s_* +*s*=-*j* (this can be demonstrated by the similar procedures in the Reference^20^ based on the above projection operator techniques of Lie groups), we can obtain the selection rule as below.

$$l_{s}=l+\sigma-s.$$

Namely, Eq. (4) in the main text.

# SI 4. The excited plasmonic scalar vortices in three spin states of SPPs

The PVL in Fig. 1(a) in the main text is illuminated by left circularly polarized vortex beams $\left| \left. \sigma,l \right\rangle\right.$ with $\sigma$ being 1 and $l$ ranging from -1 to -8. The incident light comes from the back of the PVL. Therefore, there are eight combinations of $\left( \sigma,l \right)$: (+1, -1), (+1, -2), (+1, -3), (+1, -4), (+1, -5), (+1, -6), (+1, -7) and (+1, -8). Based on Eq. (2) in the main text, the eight combinations lead to indices of irreps $j$: 0, 1, 2, 3, 4, 5, 6, and 7. Correspondingly, based on Eq. (3), topological charges of plasmonic scalar vortices in spin-0 state $l_{0}$ can take: $Mq_{0}$, $-1+Mq_{0}$, $-2+Mq_{0}$, $-3+Mq_{0}$, $-4+Mq_{0}$, $-5+Mq_{0}$, $-6+Mq_{0}$, and $-7+Mq_{0}$, which can be quickly verified by the simulation results in Fig. S2. Topological charges of plasmonic scalar vortices in spin-−1 state $l_{-1}$ can take: $1+Mq_{0}$, $Mq_{0}$, $-1+Mq_{0}$, $-2+Mq_{0}$, $-3+Mq_{0}$, $-4+Mq_{0}$, $-5+Mq_{0}$, and $-6+Mq_{0}$, which can be quickly verified by the simulation results in Fig. S3. Topological charges of plasmonic scalar vortices in spin-+1 state $l_{+1}$ can take: $-1+Mq_{0}$, $-2+Mq_{0}$, $-3+Mq_{0}$, $-4+Mq_{0}$, $-5+Mq_{0}$, $-6+Mq_{0}$, $-7+Mq_{0}$, and $-8+Mq_{0}$, which can be quickly verified by the simulation results in Fig. S4.

It is worth noting that in the main text we use right- and left-handed circularly polarized beams to excite the PVL, i.e., $\left| \left. +,0 \right\rangle\right.$ and $\left| \left. -,0 \right\rangle\right.$. Accordingly, the plasmonic vortices belonging to the $7^{th}$ and $1^{st}$ irreps are excited, respectively. The two incident beams carry no orbit angular momentum (OAM). We can also use incident beams with both SAM and OAM to excite the two irreps, e.g., $\left| \left. +,-8 \right\rangle\right.$ and $\left| \left. +,-2 \right\rangle\right.$. Take the cases of plasmonic scalar vortices in spin-0 state excited by $\left| \left. +,0 \right\rangle\right.$ and $\left| \left. +,-8 \right\rangle\right.$ as an example, as shown in Fig. 1(d) and Fig. S1(h), respectively. By comparing the two plasmonic scalar vortices, it can be observed that the plasmonic scalar vortices excited by $\left| \left. +,-8 \right\rangle\right.$ in Fig. S1(h) exhibit more topological charges, namely, when the topological charges of plasmonic scalar vortices can be controlled by the tuning OAM of incident light. By tuning the SAM of incident field can do so as well. The same story also applies to the plasmonic scalar vortices in spin-±1 states excited by incident beams with different SAM and OAM (comparing the related figures in Fig. 1(c)-(d) and Fig. S2, Fig. S3, and Fig. S4). The generation of plasmonic polarization vortices also exhibit the same properties (see Fig. S8 in SI 5).

Fig. S2. Simulated results of multiplexed plasmonic scalar vortices in spin-0 state. In the second and fourth columns of the figures, white numbers denote topological charges of multiplexed plasmonic scalar vortices.

Fig. S3. Simulated results of multiplexed plasmonic scalar vortices in spin-+1 state. In the second and fourth columns of the figures, white numbers denote topological charges of multiplexed plasmonic scalar vortices.

Fig. S4. Simulated results of multiplexed plasmonic scalar vortices in spin-−1 state. In the second and fourth columns of the figures, white numbers denote topological charges of multiplexed plasmonic scalar vortices.

# SI 5. The excited plasmonic polarization vortices

A polarization vortex can be viewed as the superposition of two scalar vortices on the circular bases, i.e.,

In the above, $|\left. + \right\rangle$ and $|\left. - \right\rangle$ denote the circular bases $\hat{L}$ and $\hat{R}$, respectively; and $\Delta\varphi$ is the phase shift between the two scalar vortices. The above equation can be recast in the following form,

where $I$ and $l_{p}$ denote the geometric and dynamic topological charges carried by the polarization vortex. They can be evaluated by following simple algebraic relations,

Substituting Eq. (3) into the above equation, we obtain,

where $q_{o}=q_{-}-q_{+}$ and $q_{e}=q_{-}+q_{+}$, where $q_{-}$ and $q_{+}$ represent the integers in plasmonic scalar vortices in spin-$\mp1$ states. Note that, when $I$ equals one, the V points can be realized. Specifically, when $I=1$ and $\Delta\varphi=2n\pi$, the V points in radially polarized SPP fields appear, namely, plasmonic polarization vortices in the cylindrical basis $\hat{\boldsymbol{\rho}}$; and when $I=1$ and $\Delta\varphi=\left( 2n+1 \right)\pi$, the V points in azimuthally polarized SPP fields appear, namely, plasmonic polarization vortices in the cylindrical basis $\hat{\boldsymbol{\varphi}}$.

On the one hand, the geometric topological charges $I$ of plasmonic polarization vortices can be quickly confirmed by checking the related Stokes fields $S_{12}=S_{1}+iS_{2}$. The Stokes parameters can be obtained by^22^,

$$\vec{S}=\left(S_{1},S_{2},S_{3}\right)=\langle\mathbf{E}_{||}|\hat{\mathbf{\sigma}}|\mathbf{E}_{||}\rangle.$$

In the above,

$$\mathbf{E}_{||}=\left(\begin{matrix} E_{+} \\ E_{-} \end{matrix}\right),\hat{\mathbf{\sigma}}=\left(\hat{\sigma}_{1},\hat{\sigma}_{2},\hat{\sigma}_{3}\right)=\left(\left(\begin{matrix} 0 & 1 \\ 1 & 0 \end{matrix}\right),\left(\begin{matrix} 0 & -i \\ i & 0 \end{matrix}\right),\left(\begin{matrix} 1 & 0 \\ 0 & -1 \end{matrix}\right)\right).$$

where $\mathbf{E}_{||}$ is the in-plane SPP fields, $E_{\pm}$ is the spin-±1 components of SPP fields, $\hat{\boldsymbol{\sigma}}$ is Pauli matrix vector. As stated in the main text, there is a simple relation between the topological charge of a polarization vortex and the one of a scalar Stokes-field vortex in $S_{12}$, $I={l_{12}}/2$, where $l_{12}$ denotes the topological charge of the scalar Stokes-field vortex^22^. The scalar Stokes-field vortex in $S_{12}$ are illustrated in Fig. S5, from which by using the above simple relation, the topological charges of multiplexed polarization vortices can be evaluated.

Fig. S5. The scalar vortices in the synthesized Stokes fields $S_{12}$ corresponding to the polarization vortices in Fig. 3(c) in the main text.

On the other hand, the dynamic topological charges $l_{p}$ of plasmonic polarization vortices can be quickly confirmed by checking the related dynamic scalar fields $\Phi$,

$$\Phi=\left(\begin{matrix} E_{x} & E_{y} \end{matrix}\right)^{T}\cdot\left(\begin{matrix} E_{x} & E_{y} \end{matrix}\right)^{T}=E_{x}E_{x}+E_{y}E_{y}=2E_{+}E_{-}.$$

As stated in the main text, there is a simple relation between the topological charge of a polarization vortex and the one of the scalar vortex in the dynamic field $\Phi$, $l_{p}={l_{\Phi}}/2$, where $l_{\Phi}$ denotes the topological charge of the scalar vortex^22^.

Besides the incident fields $|\left. + \right\rangle$ and $|\left. - \right\rangle$, the plasmonic polarization vortices in the radial, azimuthal, and in-plane SPP fields can also be excited by the incident beams $\left| \left. \sigma,l \right\rangle\right.$ with $\sigma$ being 1 and $l$ ranging from -1 to -8 (correspondingly the irreps $j=0,\ldots,7$ will be excited). For the excited polarization vortices with V points in radially and azimuthally polarized in-plane SPP field, as stated in the main text, their corresponding geometric topological charges are always 1. Their corresponding dynamic topological charges can be directly observed from Fig. S6, consistent with our obtained selection rules in Eq. (4) in the main text or Eq. here. It can also be observed that the dynamic topological charges carried by the radial and azimuthal polarization vortices are multiplexed.

Fig. S6. The plasmonic polarization vortices in the radial and azimuthal SPP fields excited by eight circularly polarized vortex beams. The order of phase singularities carried the vortices are marked by white numbers in the figures. For simplicity, only phase distributions are plotted.

The excited plasmonic polarization vortices with C points in the in-plane SPP fields are illustrated in Fig. S7, from which it can be observed that each category of plasmonic polarization vortices is a nest of multiple polarization vortices with different geometric topological charges, namely, multiplexed plasmonic polarization vortices. And the geometric topological charges carried by each plasmonic polarization vortices are consistent with the theoretical predictions of Eq. (4) in the main text. Interestingly, there are many types of polarization singularities occurs in Fig. S7, such as L lines, bright and dark C points (see Fig. S7). This observation can be further confirmed by the synthesized Stokes field $S_{12}$ (see Fig. S8 and consider the relation $I={l_{12}}/2$). The dynamic topological charges carried by the plasmonic polarization vortices can be directly observed from the dynamic scalar field $\Phi$ in Fig. S9 by considering the relation, $l_{p}={l_{\Phi}}/2$. From Fig. S9, it can be observed the dynamic topological charges carried by the plasmonic polarization vortices are multiplexed and agree well with our obtained selection rules.

Fig. S7. The multiplexed plasmonic polarization vortices in the in-plane SPP fields excited by eight circularly polarized vortex beams.

Fig. S8. The scalar vortices in the synthesized Stokes fields $S_{12}$ belonging to eight irreps of $C_{8}$ group.

Fig. S9. The multiplexed scalar vortices in the dynamic scalar fields $\Phi$ belonging to eight irreps of $C_{8}$ group.

# SI 6. Revisiting dynamic topological charges of plasmonic polarization vortices in cylindrical bases

In the above section, we obtain the selection rule for plasmonic polarization vortices in the in-plane SPP fields, whose geometric and dynamic topological charges can be obtained by Eq. . Note again that, in Eq. , when $I$ equals one, the V points can be realized. Specifically, when $I=1$ and $\Delta\varphi=2n\pi$, the polarization vortices with the V points in radially polarized SPP fields appear, namely, plasmonic polarization vortices in the cylindrical basis $\hat{\boldsymbol{\rho}}$; and when $I=1$ and $\Delta\varphi=\left( 2n+1 \right)\pi$, the polarization vortices with the V points in azimuthally polarized SPP fields appear, namely, plasmonic polarization vortices in the cylindrical basis $\hat{\boldsymbol{\varphi}}$. The presence of the radial and azimuthal plasmonic polarization vortices can be further confirmed by the transformation relationships between the circular and cylindrical bases. Next, we will revisit the dynamic topological charges of the radial and azimuthal plasmonic polarization vortices from the perspective of transformation between the circular and cylindrical bases.

An eigen vortex can be expressed in terms of circular bases, i.e., $\mathbf{E}=E_{R}\hat{R}+E_{L}\hat{L}+E_{z}\hat{z}$. Again, here, we adopt the convention for the right- and left-circular unit vectors,

$$\hat{L}=\frac{1}{\sqrt{2}}\left(\hat{x}+i\hat{y}\right),\text{ }\hat{R}=\frac{1}{\sqrt{2}}\left(\hat{x}-i\hat{y}\right).$$

At the same time, the eigen vortex can also be expressed in terms of cylindrical bases, i.e., $\mathbf{E}=E_{\rho}\hat{\rho}+E_{\varphi}\hat{\varphi}+E_{z}\hat{z}$. With the convention in Eq. , the radial and azimuthal unit vectors can be expressed as,

The cylindrical and circular bases can transform from each other,

With Eqs. and , the cylindrical components of the eigen vortex can be transformed from the circular components, i.e.,

According to Eq. (1) in the main text, the circular components of the eigne vortex can be written in a generic form,

In the above, $E_{L,q_{+}}$ and $E_{R,q_{-}}$ are the complex coefficients of partial wave components labelled by $q_{+}$ and $q_{-}$ in $E_{L}$ and $E_{R}$, respectively. Substituting Eq. into Eq. , we can obtain,

The above equation indicates that the radial and azimuthal components of the eigen vortex also exhibit a pattern of vortices as well, whose topological charges can be written as,

By further using the symmetry matching condition and cancelling irrep index $j$ in the above equation (by considering Eq. ), we can obtain the dynamic topological charges $l_{p}$ of the radial and azimuthal plasmonic polarization vortices in Eq. (4) in the main text.

# SI 7. Sample fabrications

The PVLs in this work were fabricated using a standard electron-beam lithography (EBL) and reactive ion etching (RIE) process on a glass substrate. Overview of the fabrication flow is depicted as follows.

Fig. S10 Overview of the fabrication flow.

The complete protocol is as follows:

1. Substrate Preparation: We used 10×10 mm² Borosilicate glass coverslips (thickness 0.13–0.16 mm). The substrates were sequentially cleaned in an ultrasonic acetone bath (>15 min), rinsed in hot acetone and hot IPA (5 min each), blown dry with N₂, and subsequently treated with a Piranha solution (H₂SO₄:H₂O₂ = 4:1) for 15–40 minutes to remove organic residues and hydroxylate the surface, ensuring excellent hydrophilicity.
2. Metal Deposition: A 2 nm Ti adhesion layer and a 107 nm Au layer were deposited onto the cleaned glass substrate using a BAE 370RF sputter coater. The Ti layer was deposited at 200 W for 6 s, and the Au layer was deposited at 80 W for 60 s.
3. PMMA Mask Patterning: A single layer of PMMA was spin-coated at 7000 RPM for 60 s and baked at 180 °C for 2 min. Electron-beam lithography (EBL) was performed using a 20 kV beam. The aperture patterns were exposed at a current of ~340 pA. After exposure, the sample was developed in MIBK:IPA (1:1) for 30 s, rinsed with IPA for 60 s, and dried with N₂.
4. Reactive Ion Etching (RIE): The Au layer was selectively etched through the PMMA mask using RIE. The specific etching parameters (e.g., gas chemistry, power, and pressure) were optimized to achieve the required depth. As shown in the etching rate calibration (Fig. e in the supporting information), the Au etch rate was determined to be approximately 70 nm/min, while the PMMA etch rate was only ~8 nm/min. This high selectivity (Au:PMMA ≈ 8.75:1) ensures the PMMA mask remains intact throughout the 1.5-minute etching process required to fully remove the 107 nm Au layer.
5. Characterization: The final etched structures were characterized using AFM to measure the step height and surface morphology at different etching intervals (1, 2, 3, 4 min).

It is worth noting that our fabricated sample is sensitive to fabrication defects. Following we discuss the effects of metal thickness, sidewall roughness, and etch width on the sample.

1. Metal thickness (2 nm Ti + 107 nm Au): 107 nm was chosen to ensure sufficient opacity in the visible/NIR range while balancing depth and lateral resolution for near-field detection. Although alternative thicknesses (e.g., 200 nm in prior literature) are viable, thickness variations alter the relative amplitudes of different plasmonic vortex orders. We specifically selected 107 nm to enable the detection of a broader range of vortex modes.
2. Sidewall roughness: Given the high etching selectivity (Au ~70 nm/min vs. PMMA ~8 nm/min), the sidewalls are expected to be relatively vertical, implying the performance of the sample will be less affected.
3. Etch width (slit width): Similar to thickness, the slit width influences the relative amplitudes of the different orders of plasmonic vortices. Through simultaneous finite-element simulations optimizing both thickness and width, we selected a slit width of 200 nm to maximize the number of detectable vortex orders.

# SI 8. Experimental setup and verifications

The optical measurements were conducted on a homemade spin-selective and phase-resolved scanning near-field optical microscope (SSPR-SNOM), as illustrated in Fig. S11. A continuous laser wave was directed into a beam splitter (BS) to generate signal and reference optical paths with adjustable power. The frequency shift ΔΩ between the two paths was fixed by selecting the same diffraction order of acousto-optic modulators. Circular polarization was employed to excite the sample in the signal path, achieved through a linear polarizer (LP), a half-wave plate (HWP), and a quarter-wave plate (QWP). The focused beam was then directed onto the surface of plasmonic vortex lenses (PVLs) using a 10× objective lens (NA = 0.3). The sample position and beam center were imaged using a light source and a CCD. Near-field plasmonic vortices were coupled using an aperture probe, which is sensitive to the electric field components in the plane. The probe-sample distance was precisely controlled at the nanoscale. Simultaneously, a Grin Lens was employed in the reference path to efficiently couple the light beam after polarization modulation, forming an interference profile with the signal component. This can be expressed as an equation

where $\Delta\phi$ represents the relative phase and the last term denotes the projection of the signal polarization $\hat{e}_{\text{sig}}$. By selectively controlling the spin state in the reference $\hat{e}_{\text{ref}}$, the corresponding left and right circular components were collected. Finally, the relative amplitude and phase were detected by a photomultiplier tube (PMT) and visualized using a lock-in amplifier (LIA) based on the frequency shift ΔΩ. The distribution of plasmonic polarization vortices was obtained by combining the two orthogonal components.

Fig. S11. Experimental setup of SNOM system. The incident laser beam was split into signal and reference paths using a beam splitter (BS). Both paths underwent modulation with an acousto-optic modulator (AOM) and precise polarization control using a linear polarizer (LP), half-wave plate (HWP), and quarter-wave plate (QWP) to generate a circularly polarized beam. The beam was focused onto plasmonic vortex lenses (PVLs) using a 10x objective lens (NA = 0.3). A light source and CCD camera facilitated imaging. The near-field profile interfered with the reference beam through a fiber coupler, detected by a photomultiplier tube (PMT), and demodulated using a lock-in amplifier (LIA) for analysis.

To further explain the discrepancies discussed in the main text, we carried out an additional verification experiment without the sample, in which only the optical power in the signal arm was intentionally varied while all other experimental conditions remained unchanged. The heterodyne interference signal was then monitored using an oscilloscope. As shown *Fig. S12*, the yellow sinusoidal waveform corresponds to the 20 kHz frequency shift introduced by the acousto-optic modulator (ΔΩ=20 kHz), whereas the green waveform represents the AC heterodyne interference signal. After reducing the optical power in the signal arm, the peak-to-peak voltage of the green waveform decreased from approximately 1.06 V to 610 mV, directly demonstrating that the amplitude of the AC interference term decreases significantly as the signal intensity becomes weaker.

This effect becomes even more pronounced in the plasmonic vortex measurements. For example, the region corresponding to $l_{s}=-10$ possesses a relatively strong field amplitude and therefore produces a sufficiently strong heterodyne signal, allowing the lock-in amplifier to recover the full 2π phase variation. By contrast, as the measurement approaches the vortex center, particularly in the regions corresponding to $l_{s}=-2$ and $l_{s}=+6$, the optical field amplitude decreases substantially. The resulting weaker heterodyne signal leads to reduced phase-demodulation accuracy, giving rise to the incomplete phase coverage observed in the experimental results.

Fig. S12. Experimental verification of the dependence of the heterodyne interference signal on the signal-arm optical amplitude. (a) Oscilloscope traces with a relatively strong signal-arm optical field, where the peak-to-peak voltage of the green heterodyne interference signal is approximately 1.06 V. (b) Oscilloscope traces after reducing the optical intensity in the signal arm, where the peak-to-peak voltage of the green heterodyne interference signal decreases to approximately 610 mV. The yellow sinusoidal waveform in both panels represents the electrical driving signal of the acousto-optic modulator (AOM), corresponding to the heterodyne frequency shift of 20 kHz.

The incident light is right-handed circularly polarized beam, $|\left. -,0 \right\rangle$. The near-field distributions of the plasmonic scalar vortex in spin-$-1$ state are also measured, as illustrated in *Fig. S13*. Comparing *Fig. S13* (a) and (b) with Fig. 1(c) denoted by $s=-1$ in the main text, it can be found that the measured and simulated near-field results also agree well with each other. The theoretical and measured phase distributions along a closed path in *Fig. S13* (b) are illustrated in *Fig. S13* (c), showing the good consistency as well even though there are some discrepancies, which can be explained by the similar reasons discussed above and in the main text..

Fig. S13. Illustration of the plasmonic scalar vortex in spin-−1 state in near-field region: (a) the magnitude and (b) the phase distributions of experimentally measured results; (c) plots the simulated and experimental phase distributions of the vortex along a closed contour denoted by magenta color in (b).

# SI 9. Demonstrations in the cases of C2 and C3 group symmetries

Fig. S14 An illustration of the PVL with 2-fold rotational symmetries and the simulated magnitude and phase patterns of the spin-0 state of SPPs on a cut plane above the PVL surface $\left( z=150 nm \right)$. In (a), the top view of the PVL is shown. (b)-(c) correspond to the electric field patterns excited by the left circularly polarized incident vortex beam with orbit quantum number l ranging from -1 to -2. In the first and third columns, the zoomed-in phase patterns are shown in the lower panels. They correspond to the phase distributions of the central parts enclosed by the white solid circles in the upper panels. In the second and forth columns shaded by the grey color, the upper and the lower panels depict the magnitude and the phase at the very center of the PVL. The correspondences are marked by the green arrows in the figure.

This part aims to re-verify our theory in the PVLs with $C_{2}$ and $C_{3}$ group symmetries. For the sake of simplicity, next we only show the plasmonic scalar vortices in the out-of-plane SPP fields, namely, focusing on the spin-0 state of SPPs.

Firstly, let’s take a look at the case of the $C_{2}$ group. Keeping the same parameters as for the PVL with $C_{8}$ group symmetries in the main text, we design a PVL with $C_{2}$ group symmetries, as shown in Fig. S14(a). The $C_{2}$ group has only two irreps with odd and even parities, respectively. As a result, the eigenmodes of the PVL are either symmetric or anti-symmetric. The PVL is illuminated by the left circularly polarized vortex beams with *l* ranging from -1 to -2. According to Eq. (3) in the main text, the two combinations of (*σ*, *l*), i.e., (+1, -1) and (+1, -2), correspond to *j*=0 and 1, respectively. From the irreps of the $C_{2}$ group (substituting *M*=2 into Table S1), it can be observed that the eigenmodes belonging to the 0^th^ and the 1^st^ irreps correspond to the even and odd parities, respectively. We simulate the excited plasmonic electric fields under the same simulation setup. The magnitude and phase patterns of the spin-0 state are displayed in Fig. S14. From Fig. S14(b), it can be observed that besides the plasmonic vortex with the topological charge of -2 exhibiting in the outer ring-like region, a plasmonic mode with even parity (the trivial plasmonic vortex mode carrying zero topological charge) is formed in the central region. From Fig. S14(c), it can be observed that besides the plasmonic vortex with the topological charge of -3 exhibiting in the outer region, a plasmonic mode with odd parity is formed in the central region instead of a plasmonic vortex.

Fig. S15 An illustration of the PVL with 3-fold rotational symmetries and the simulated magnitude and phase patterns of the spin-0 state of SPPs on a cut plane above the PVL surface $\left( z=150 nm \right)$. In (a), the top view of the PVL is shown. (b)-(d) correspond to the field patterns excited by the left circularly polarized incident vortex beam with orbit quantum number l ranging from -1 to -3. The zoomed-in phase patterns are shown in the lower panels. They correspond to the phase distributions of the central regions enclosed by the white solid circles in the upper panels.

We further investigate a PVL with $C_{3}$ group symmetries, as shown in Fig. S15(a). The $C_{3}$ group has three irreps (substituting *M*=3 into Table S1). The PVL is illuminated by the left circularly polarized vortex beams with orbit quantum number ranging from -1 to -3. Therefore, there are three combinations of (*σ*, *l*), i.e., (+1, -1), (+1, -2) and (+1, -3). Based on Eq. (3) in the main text, the three combinations correspond to *j*=0, 1, 2. Therefore, the topological charges in the central region are *l*_0_=0, -1, +1, and the topological charges in the outer region are $l_{0}+3q_{0}$. Keeping the same simulation setup as in the main text and in the previous section, the magnitude and phase patterns of the spin-0 state are simulated, as displayed in Fig. S15. From Fig. S15 (b)-(d), it can be observed that the excited plasmonic fields indeed form the vortex modes carrying the topological charges of 0, -1 and +1, respectively, in the central regions enclosed by the white dashed circles. In the outer ring-like regions (outside the white dashed circles), it can be seen that there exist the higher-order plasmonic vortices with the topological charges which equal the topological charges in the central regions plus $3q_{0}$, as denoted by white numbers in Fig. S15. The simulation results of the PVL with symmetries composing the $C_{2}$ and the $C_{3}$ groups well verify our theory.

# SI 10. Demonstration in microwave plasmonic structure

we have performed an additional numerical simulation for the sun-flower-like metallic structure depicted in Fig. S1(vi) (also see Fig. S16a), in order to verify the universality of the proposed theory. The analysis procedure and obtained results are summarized as follows:

Fig. S16. Sun-flower-like metallic structure: (a) Illustration of the structure and excitation configuration; (b) Projection operator technique for projecting the dipole radiation field; (c) The metallic structure made of copper (the 1st layer) and the external excitation system (from 2nd to 5th layers).

1. Since the structure is metallic and operates in the microwave regime, it can be modeled as a PEC. In this regime, the extended theoretical framework described above must be employed to analyze its electromagnetic scattering properties.

2. Using the projection operator technique from group theory, we can rigorously determine which incident field configurations can excite specific eigenmodes of the structure. The relevant derivations closely follow those presented in SI 1.2, SI 1.3, and SI 2. In the microwave regime, standard Laguerre–Gaussian beams are not readily available as excitation sources. Instead, microwave experiments and simulations typically employ dipole antennas, microstrip lines, or similar feeds. Here, we adopt a short microstrip line as the excitation source, which can be well approximated as a small dipole. The excitation geometry is illustrated in Fig. S16b. We also apply the projection operator technique in Eqs. (16)-(17) of the revised supporting information, i.e.,

$$\hat{P}^{j}\left\{\mathbf{E}_{dipole}\right\}\neq0.$$

Or,

$$\hat{P}^{j}\left\{\mathbf{E}_{dipole}\right\}=\mathbf{E}_{dipole}.$$

Applying the projection operator technique corresponding to the $A_{1}$ irreducible representation to this dipole incident field yields a nonzero projected component (i.e., Eq. Fig. S16 is satisfied), as shown in Fig. S16b. This indicates that the dipole source can indeed excite the eigen-electric-field patterns belonging to the $A_{1}$ irreducible representation.

3. Based on this analysis, we design a five-layer device, as shown in Fig. S16, to practically realize the excitation of the $A_{1}$ irreducible representation of the flower-like structure using a dipole feed. The first layer of the device is the sun-flower-like metallic structure with the $D_{8}$ group symmetry. The structure is etched on a dielectric plate made of Rogers 4530B (with the relative permittivity 3.48, the loss tangent 0.0037 and the thickness 0.508 mm), which serves as the second layer. The same dielectric plate is used for the third and the fifth layers. The fourth layer is made of metal film and serves as the ground of the device. Two microstrip lines are etched on the lower surface of the fifth layer and the upper surface of the third layer, respectively, and are connected by a via from the third to fifth layer, as shown in Fig. S16c. The third, fourth and fifth layers establish the feeding system which can be engineered to generate the required incident field to excite the structure. Due to the existence of the metal ground (the fourth layer), the incident field is dominated by the field radiated by the microstrip line on the upper surface of the third layer (see Fig. S16c).

4. The excited electric field components are presented in Fig. S16(a) and (b). Specifically, the $E_{z}$ component (see Fig. S16a) exhibits a scalar vortex of order 0. The in-plane components $E_{x}$ and $E_{y}$ (see Fig. S16b) form a polarization vortex carrying a topological charge of 1, consistent with Eq. (4) in our revised main text. When combined, these $E_{z}$ and in-plane field components produce a skyrmion-like vector configuration, as shown in Fig. S16c.

Fig. S17. Excited electric-field multidimensional vortex configurations at 3.77 GHz: (a) Amplitude and phase distributions of $E_{z}$; (b) In-plane electric field amplitude and polarization distribution; (c) EM skyrmion synthesized with the normalized real part of the three-component electric field.

# SI 11. Symmetries of pentagonal plasmonic quasicrystals

**Table S2.** Irreps of the $D_{5}$ group. In the table, $m$ ranges from 0 to 4, and $\varepsilon=e^{\frac{i\varphi}{M}}$, where $M=5$ and $\varphi$ denotes the azimuthal angle of polar coordinate. The topological charges carried by the scalar vortices in spin-$s$ state are marked by $l_{s}$, by which the OAMs of the vortices can be evaluated as $l_{s}\hbar$.^23^

| $D_{8}$ | *j* | *r^m^* | $g\circ r^{m}$ | Scalar vortex in spin- $s$ state |
| --- | --- | --- | --- | --- |
| *A*_1_ | *j*=0 | $\left( \varepsilon^{j} \right)^{m}$ | $\left( \varepsilon^{j} \right)^{m}$ | $l_{s}=-s+Mq_{s}$ |
| *A*_2_ | *j*=0 | $\left( \varepsilon^{j} \right)^{m}$ | $-\left( \varepsilon^{j} \right)^{m}$ | $l_{s}=-s+Mq_{s}$ |
| *E*_1_ | *j*=1 | $\left( \varepsilon^{j} \right)^{m}$ | 0 | $l_{s}=-1-s+Mq_{s}$ |
|  | *j*=4 | $\left( \varepsilon^{j} \right)^{m}$ | 0 | $l_{s}=-4-s+Mq_{s}$ |
| *E*_2_ | *j*=2 | $\left( \varepsilon^{j} \right)^{m}$ | 0 | $l_{s}=-2-s+Mq_{s}$ |
|  | *j*=3 | $\left( \varepsilon^{j} \right)^{m}$ | 0 | $l_{s}=-3-s+Mq_{s}$ |

The plasmonic pentagonal PVL in the main text hold 5-fold rotation and 5-fold reflection symmetries. The rotations and the reflections form a rosette group, i.e., the $D_{5}$ group^19^. The irreducible representations (irreps) of the $D_{5}$ group are given in Table S2. The generator of rotation operations in $D_{5}$ group is denoted by $r$, which is a rotation of an angle $\theta_{0}={2\pi}/5$ with respect to the rotation axis (e.g., the $z$ axis here). All rotations of the $D_{5}$ group can be written in a set of $\left\{ 1,r,\ldots,r^{4} \right\}$. The generator of reflection operations in $D_{5}$ group is denoted by $g$, where $g$ is a reflection with respect to a reflection plane (the plane makes an angle of ${\theta_{0}}/2$ with respect to the $xoz$ plane). All reflections of the $D_{5}$ group can be written in a set of $\left\{ g,g\circ r,\ldots,{g\circ r}^{4} \right\}$, where ${g\circ r}^{m}$ reprensents a reflection whose reflection plane makes an angle of $\left( \pi-\frac{m-1}{2}\theta_{0} \right)$ with respect to the $xoz$ plane, and *◦* denotes the group product. The $D_{5}$ group is the union of the above two sets. According to the group representation theory, the $D_{8}$ group contains 4 irreps, including two nondegenerate or one-dimensional ones marked by $A_{1}$ and $A_{2}$, and two doubly degenerate or two-dimensional marked by $E_{1}$ and $E_{2}$. In Table S2, an irrep index is used, denoted by $j$, which originates from the irrep index of $C_{5}$ group since the $D_{5}$ group is the direct product of the $C_{5}$ group and the $D_{1}$ group, i.e., $D_{5}=C_{5}\otimes D_{1}$, where $D_{1}$ group is the reflection group isomorphic to the $C_{2}$ group^19^. The $A_{1}$ and $A_{2}$ irreps are indexed by the same $j=0$. The scalar basis function of each irrep can be a scalar vortex in spin-$s$ state, whose topological charges $l_{s}$ are linked with the irrep indices $j$ (see Eq. (1) in the main text). Such the symmetry analysis can be readily extended to a general case of arbitrary-order quasicrystal symmetries^24^.

**Reference**

1.	Dai, Y. *et al.* Plasmonic topological quasiparticle on the nanometre and femtosecond scales. *Nature* **588**, 616–619 (2020).

2.	Spektor, G. *et al.* Revealing the subfemtosecond dynamics of orbital angular momentum in nanoplasmonic vortices. *Science* **355**, 1187–1191 (2017).

3.	Jin, Z. *et al.* Phyllotaxis-inspired nanosieves with multiplexed orbital angular momentum. *ELight* **1**, 1–11 (2021).

4.	Yang, Y., Thirunavukkarasu, G., Babiker, M. & Yuan, J. Orbital-angular-momentum mode selection by rotationally symmetric superposition of chiral states with application to electron vortex beams. *Physical Review Letters* **119**, 094802 (2017).

5.	van Nielen, N. *et al.* Electrons Generate Self-Complementary Broadband Vortex Light Beams Using Chiral Photon Sieves. *Nano Lett.* **20**, 5975–5981 (2020).

6.	Wang, H. *et al.* Plasmonic vortex generator without polarization dependence. *New J. Phys.* **20**, 033024 (2018).

7.	Yue, F. *et al.* Vector vortex beam generation with a single plasmonic metasurface. *ACS photonics* **3**, 1558–1563 (2016).

8.	Cognée, K. G., Doeleman, H. M., Lalanne, P. & Koenderink, A. F. Generation of Pure OAM Beams with a Single State of Polarization by Antenna-Decorated Microdisk Resonators. *ACS Photonics* **7**, 3049–3060 (2020).

9.	Ni, J. *et al.* Giant helical dichroism of single chiral nanostructures with photonic orbital angular momentum. *ACS nano* **15**, 2893–2900 (2021).

10.	Tang, B., Zhang, B. & Ding, J. Generating a plasmonic vortex field with arbitrary topological charges and positions by meta-nanoslits. *Appl. Opt., AO* **58**, 833–840 (2019).

11.	Gao, L. *et al.* Generating plasmonic vortex field with spin-dependent metananoslots. *J. Opt. Soc. Am. B, JOSAB* **37**, 2179–2184 (2020).

12.	Heeres, R. W. & Zwiller, V. Subwavelength Focusing of Light with Orbital Angular Momentum. *Nano Lett.* **14**, 4598–4601 (2014).

13.	Arikawa, T. *et al.* Transfer of orbital angular momentum of light to plasmonic excitations in metamaterials. *Science Advances* **6**, eaay1977 (2020).

14.	Chew, W. C., Tong, M. S. & Hu, B. *Integral Equation Methods for Electromagnetic and Elastic Waves*. (Springer International Publishing, Cham, 2009). doi:10.1007/978-3-031-01707-0.

15.	Zheng, X. *et al.* On the Use of Group Theory in Understanding the Optical Response of a Nanoantenna. *IEEE Transactions on Antennas and Propagation* **63**, 1589–1602 (2015).

16.	Kuppe, C. *et al.* Measuring optical activity in the far-field from a racemic nanomaterial: diffraction spectroscopy from plasmonic nanogratings. *Nanoscale Horiz.* **4**, 1056–1062 (2019).

17.	Collins, J. T. *et al.* Enantiomorphing Chiral Plasmonic Nanostructures: A Counterintuitive Sign Reversal of the Nonlinear Circular Dichroism. *Advanced Optical Materials* **6**, 1800153 (2018).

18.	Serre, J.-P. *Linear Representations of Finite Groups*. vol. 42 (Springer, 1977).

19.	Dresselhaus, M. S., Dresselhaus, G. & Jorio, A. *Group Theory: Application to the Physics of Condensed Matter*. (Springer Science & Business Media, 2007).

20.	Yang, J. *et al.* Polarization singularities in planar electromagnetic resonators with rotation and mirror symmetries. *Photonics Research* https://doi.org/10.1364/PRJ.485625 (2023) doi:10.1364/PRJ.485625.

21.	Yang, J. *et al.* Customizing the Topological Charges of Vortex Modes by Exploiting Symmetry Principles. *Laser & Photonics Reviews* **16**, 2100373 (2022).

22.	Bliokh, K. Y., Alonso, M. A. & Dennis, M. R. Geometric phases in 2D and 3D polarized fields: geometrical, dynamical, and topological aspects. *Reports on Progress in Physics* **82**, 122401 (2019).

23.	Yang, J., Jiang, J., Wang, J. & Zheng, X. Singular Electromagnetics: From Phase Singularities to Optical Skyrmions and Beyond. *Advanced Physics Research* **n/a**, 2400083.

24.	Yang, J. *et al.* Optical Singularities in Photonic Microstructures with Rosette Symmetries: A Unified Theoretical Scheme. *Engineering* **45**, 59–69 (2025).
